# The explant developmental stage profoundly impacts small RNA-mediated regulation at the dedifferentiation step of maize somatic embryogenesis

**DOI:** 10.1038/s41598-019-50962-y

**Published:** 2019-10-10

**Authors:** Vasti T. Juárez-González, Brenda A. López-Ruiz, Patricia Baldrich, Eduardo Luján-Soto, Blake C. Meyers, Tzvetanka D. Dinkova

**Affiliations:** 1 Departamento de Bioquímica, Facultad de Química, Universidad Nacional Autónoma de México, CDMX, 04510 México; 2 Donald Danforth Plant Science Center, 975 North Warson Road, St. Louis, MO 63132 USA; 3 Division of Plant Sciences, University of Missouri, Columbia, Missouri 65211 USA

**Keywords:** Non-coding RNAs, Plant biotechnology

## Abstract

Maize somatic embryogenesis (SE) requires the induction of embryogenic callus and establishment of proliferation before plant regeneration. The molecular mechanisms underlying callus embryogenic potential are not well understood. Here we explored the role of small RNAs (sRNAs) and the accumulation of their target transcripts in maize SE at the dedifferentiation step using VS-535 zygotic embryos collected at distinct developmental stages and displaying contrasting *in vitro* embryogenic potential and morphology. MicroRNAs (miRNAs), *trans*-acting siRNAs (tasiRNAs), heterochromatic siRNAs (hc-siRNAs) populations and their RNA targets were analyzed by high-throughput sequencing. Abundances of specific miRNAs, tasiRNAs and targets were validated by qRT-PCR. Unique accumulation patterns were found for immature embryo at 15 Days After Pollination (DAP) and for the callus induction from this explant, as compared to 23 DAP and mature embryos. miR156, miR164, miR166, tasiARFs and the 24 nt hc-siRNAs displayed the most strikingly different patterns between explants and during dedifferentiation. According to their role in auxin responses and developmental cues, we conclude that sRNA-target regulation operating within the 15 DAP immature embryo explant provides key molecular hints as to why this stage is relevant for callus induction with successful proliferation and plant regeneration.

## Introduction

Plant zygotic embryogenesis is determined by complex gene regulatory networks to achieve appropriate spatiotemporal cell division and differentiation^1–3^. An alternative gene expression program, promoted by high phytohormone concentrations, allows the accomplishment of embryogenesis and plant regeneration from somatic cells. This process is known as Somatic Embryogenesis (SE)^4,5^. The required conditions and regulatory programs vary between plant species. For maize, embryogenic callus induction and plant regeneration success strongly depends on the genotype and tissue used as explant^6,7^.

Gene regulation by small RNAs (sRNAs), as revealed by differential abundance analyses, has emerged as an important aspect of SE induction in a variety of plant species^8–10^ including maize^11–13^. sRNA silencing mechanisms are involved in genome stability, defense against virus and transposable elements (TEs), epigenetic modifications, and post-transcriptional regulation^14,15^. microRNAs (miRNAs) and small interfering RNAs (siRNAs) distinguish by their biogenesis pathways^16^. The siRNA class is further sub-divided in heterochromatic siRNAs (hc-siRNAs), secondary siRNAs and natural anti-sense siRNAs (natsiRNAs)^17^. Secondary siRNAs comprise phased siRNAs (phasiRNAs) and *trans*-acting siRNAs (tasiRNAs).

miRNAs are derived from single strand RNAs (ssRNAs) transcribed by RNA polymerase II that fold into stable stem-loop secondary structures called primary miRNAs (pri-miRNAs). The double-stranded RNA (dsRNA) portion of a pri-miRNA is recognized by a DICER-LIKE (DCL) enzyme, DCL1 in plants, to generate the miRNA precursor (pre-miRNA) in the nucleus and subsequently a small RNA duplex (20 to 22 nt) with a two nucleotide 3′ end overhang^15^. siRNAs arise from dsRNA precursors that result from the action of RNA-DEPENDENT RNA POLYMERASES (RDRs), a group of enzymes originally identified in viruses by their role during the viral genome replication^18^. Different DCL family members, DCL2, DCL3 or DCL4 in *Arabidopsis thaliana*, process dsRNA^17^. While hc-siRNAs are processed by DCL3, secondary siRNAs usually require the action of DCL4. The length of siRNAs (20–24 nt) depends on the particular DCL enzyme used in their production. Both miRNA and siRNA duplexes are stabilized through HUA ENHANCER 1 (HEN1)-dependent methylation at their 3′ ends^19^. sRNA loading on specific ARGONAUTE (AGO) proteins yields a functional RNA-Induced Silencing Complex (RISC), with AGO specificity further diversifying sRNA roles based on mature size, 3′ end nucleotide identity, and the specific silencing mechanism used on targets^15,19^.

Among the ten different AGO proteins present in Arabidopsis, AtAGO1 loads miRNAs and phasiRNAs, AtAGO10 specifically loads miR165/166, AtAGO7 loads a unique miRNA (miR390) involved in tasiRNA biogenesis from *TAS3*, AtAGO2 functions in antiviral defense, and the AGO4 clade (AGO4, AGO6, AGO9) functions in silencing of transposons and repeated sequences through a specific RNA-directed DNA methylation mechanism, RdDM^20,21^. The major AGO clades defined in Arabidopsis have been discovered in other plant species, including rice and maize^20^. However, the genomes of many plant species encode additional AGO family members.

To link the role of sRNAs with gene regulation of particular developmental processes, mutants for the enzymes required in their biogenesis and function have been characterized^22,23^. In maize, a few mutants are available for components of tasiRNA and RdDM pathways^24–26^. However, their genotype is poor for SE induction, and some display sterility^27^. We have reported the behavior of miRNAs and other sRNAs during maize SE using immature embryos (IE) at 15 days after pollination (DAP) in the highly embryogenic genotype, Tuxpeño VS-535^12,13^. However, the impact of different zygotic embryo developmental stages on sRNAs patterning during dedifferentiation was not explored. Here we characterized callus induction of three different explants: immature embryos at 15 DAP (IE15), as a standard tissue used for SE induction in VS-535; at 23 DAP (IE23), outside of the usual development window to induce embryogenic callus, and mature embryo (ME), known as non-embryogenic tissue. Contrasting phenotypes were observed between explants, induced calli, and their embryogenic potential. By high-throughput sequencing of the sRNAs and transcripts from each explant/induced callus tissue in duplicated biological samples, we found major changes in the different sRNA populations, depending on the developmental stage of the explant. Although sRNA readjustments during callus induction showed similar overall patterns for all explants, IE15 displayed differential accumulation of specific miRNAs and tasiRNAs, as well as their targets, providing molecular hints as to why this stage is relevant for successful embryogenic callus induction. On the other hand, the abundance of 24 nt hc-siRNAs increased in explants of later developmental stages (IE123 and ME) when compared to the IE15. Since the process of dedifferentiation is accompanied by a substantial reduction of this sRNA class, we propose that the initial levels in the explant might also impact the success of embryogenic callus induction.

## Results

### The embryogenic potential of induced calli depends on the developmental stage of the maize embryo

**Figure 1:**
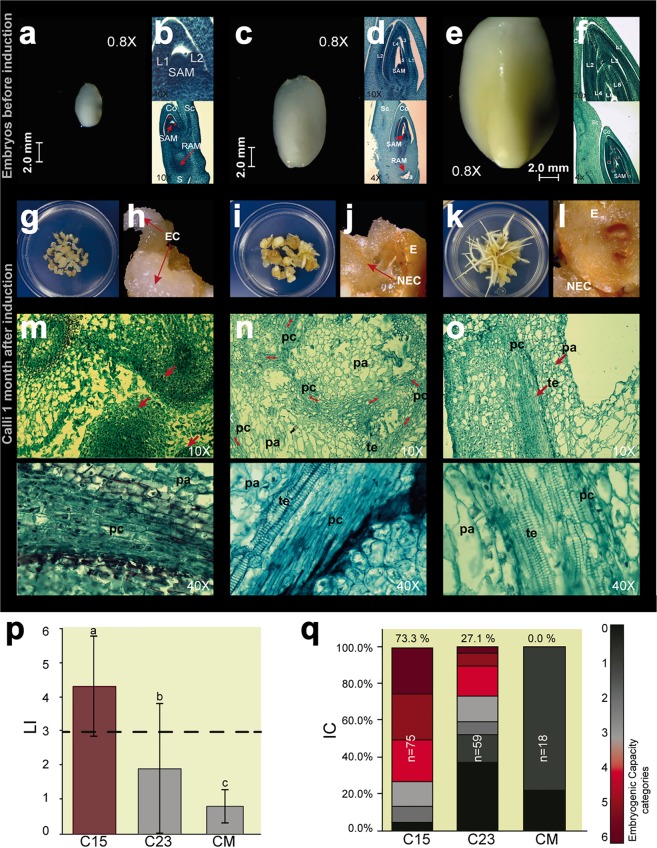
Morphological and histological characterization of callus induction using embryos collected at different developmental stages. (**a**) Immature Embryo at 15 days after pollination; IE15. (**c**) Immature Embryo at 23 days after pollination; IE23. (**e**) Mature Embryo; ME. (**b**,**d**,**f**) Histological analysis of longitudinal sections across IE15, IE23 and ME, respectively. (**g**,**i**,**k**) Callus from IE15, IE23 and ME, respectively, at one month of induction. (**h**,**j**,**l**) Callus morphology from IE15, IE23 and ME, respectively (C15, C23 and CM), observed at one month of induction by stereoscopic microscopy. (**m**–**o**) Histological analysis of callus cross-sections from IE15, IE23 and ME, respectively, at one month of induction. (**p**) Line index (LI) bar plot. The dotted line represents the minimum LI value of an embryogenic batch (**q**) *In vitro* Capacity (IC) bar plot. Embryogenic categories are designated with the red color palette; grayscale palette represents the non-embryogenic categories. L1-L5: Leaf primordia in the maize embryo; Co: Coleoptile; Sc: Scutellum; SAM: Shoot Apical Meristem; RAM: Root Apical Meristem; S: suspensor; Ca: Calyptra; E: Embryo; EC: Embryogenic Callus; NEC: Non-Embryogenic callus; te: tracheary elements; pc: procambium cells; pa: parenchyma cells.

To assess the relationship between the embryo developmental stage and its *in vitro* embryogenic potential, we compared the morphology of IE15, IE23 and ME and to one-month induced callus from each tissue (Fig. 1). IE23 has double the size of IE15 (Fig. 1c vs. 1a), while ME represents a later developmental stage showing onset of desiccation effects according to its yellowing color (Fig. 1e). In addition, while IE15 displayed one or two established leaves^2^ (L1-L2; Fig. 1b), IE23 had four or five leaves (L4-L5; Fig. 1d) and ME showed six-leaf primordia (Fig. 1f). Furthermore, IE23 showed degeneration of the suspensor (marked with “S” in IE15) and the presence of calyptra behind the root apical meristem (RAM; Fig. 1d, lower section). The ME had larger, better-organized scutellum cells indicating full embryo development^2^. Such differences between all embryos were consistent across sections obtained from distinct samples.

Embryogenic callus was readily detected in tissues induced from IE15 during the first days of induction and it showed continuous proliferation. After one month of subculture, these tissues (calli from IE15 embryos, henceforth “C15”) displayed full degeneration of the original explant and mostly consisted of embryogenic and non-embryogenic calli (Fig. 1g,h). The main phenotypic response of IE23 to dedifferentiation (calli from IE23 embryos, henceforth “C23”) included swelling of the embryo scutellum, thickening in the posterior embryo axis and non-friable calli formation on the side of the embryo in contact with the medium (Fig. 1i,j). The ME explant displayed the most contrasting response (calli from ME embryos, henceforth “CM”), characterized by the appearance of a small proportion of non-friable calli and massive root generation through germination (Fig. 1k,l). All tissues showed early vascular organization, represented by procambial and parenchymatic cells (Fig. 1m–o; lower sections), similar to what was observed in early xylem and protoxylem (tracheal elements presence)^28^. Interestingly, the protoxylem appeared erratic and distributed across the tissues (Fig. 1n, upper section). In contrast to C23 and CM, C15 displayed particular accumulation of small, isodiametric and densely packed cells (Fig. 1m, upper section), common for meristems^29^.

The embryogenic responses were evaluated according to the average Line Index (LI)^7^, obtained by observation of calli on different embryo batches at one month after induction (Methods and Table S1). Embryogenic responses (LI ≥ 3) were observed only for C15 (LI = 4.3; Fig. 1p). The mean LIs for C23 and CM were 1.9 and 0.8, respectively. Furthermore, the three batches presented significant differences in the LI score according to the Induction Capacity (IC), which calculates the proportion of embryos with embryogenic response in each batch. Seventy-three percent of IE15 generated embryogenic calli, while only 27.1% of IE23 formed this type of callus. No embryogenic response was observed for ME (IC = 0.0%; Fig. 1q). Based on these observations, we confirmed that embryogenic callus formation in the maize VS-535 genotype depends on a narrow zygotic embryo developmental stage. The *in vitro* embryogenic potential sharply drops for 23 DAP embryo, leaf stage four^2^ (L4; Fig. 1d) and is null upon completion of development (L6; Fig. 1f). Further follow-up on callus subculture confirmed substantial differences between C15, C23 and CM phenotypes (Fig. S1a) and proliferation rates (Fig. S1b). C23 and CM displayed non-embryogenic tissues with high oxidation levels proliferating at significantly lower rates than C15 since early subcultures. In addition, only C15 was able to regenerate plants (Fig. S1c).

### Small RNAs during embryo development and callus induction

**Figure 2:**
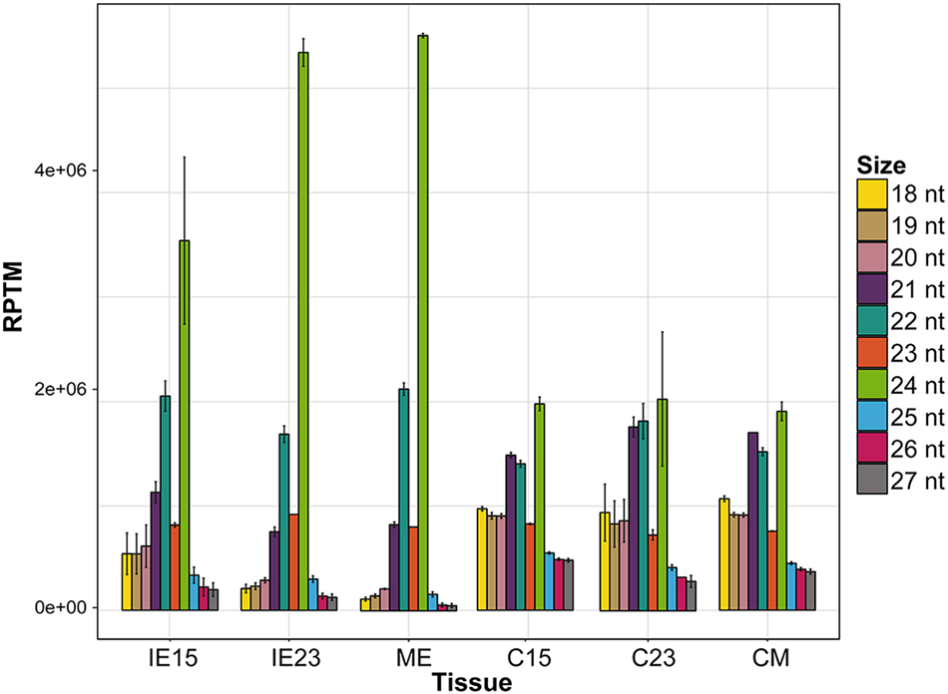
sRNA library size distribution in the different maize embryos and induced callus tissues. Size distribution is shown by normalized genome-matched reads (Reads Per 10 Million, RPTM). Mean values of the two biological replicates with standard deviation were used. IE15: Immature Embryos 15 days after pollination; IE23: Immature Embryos 23 days after pollination; ME: Mature Embryos; C15, C23 and CM: Callus tissues at one month after induction from IE15, IE23, and ME, respectively.

To explore how sRNAs could impact the contrasting dedifferentiation response of developmentally distinct zygotic embryos, the six previously characterized tissues (IE15, IE23, ME, C15, C23, and CM) were used for sRNA analysis in two biological replicates. Over 13 million sRNAs (18 to 30 nt) were sequenced per library, of which ~62% (54.6 to 66.7%) matched to the maize B73 genome (Table S2). sRNAs of 21, 22 and 24 nt were the most abundant in all tissues (Table S2; Fig. 2). However, their distribution patterns varied among tissues as follows (Figs 2 and S2): (1) In zygotic embryos, the 24-nt sRNAs were the most abundant population, increasing substantially towards embryo maturation (up to the 55.5% of ME reads), followed by 22- and 21-nt sRNA populations; (2) The 21-nt sRNA proportion was greater in calli than in embryo for all developmental stages; (3) 22 nt abundances were relatively unchanged in all tissues; (4) A noticeable decrease in 24-nt sRNAs was observed upon callus induction for all developmental stages, resulting in similar proportions of 21, 22 and 24 nt sRNAs. Overall, these results support the occurrence of sRNA length readjustment due to the dedifferentiation process, independent of the explant developmental stage or the calli embryogenic potential. However, in spite of the similar sRNA length distribution in all calli tissues, particular sRNA classes and families might differ in their accumulation patterns.

### Differential accumulation of miRNAs

Since dedifferentiation in SE involves major reprogramming in response to external *in vitro* stimuli, and miRNAs are essential regulators in developmental and stress responses, we examined miRNA levels in our data. Members of 28 known miRNA families were identified considering all libraries (Table S3). Since miRNAs from the same family showed similar patterns between tissues, with some exceptions, we merged them, combined and normalized (to reads per ten million; RPTM) their abundances for subsequent analyses. Clustering by the RPTM values in different tissues rendered groups with high (339 to 73188 median RPTM) and low (0 to 312 median RPTM) abundances across all samples (Fig. S3; Table S3). For example, the highly abundant miR159, miR167, miR156, miR319 and miR166 families, comprised one of the clusters, whereas the less abundant miR397, miR1432, miR399, miR2118, miR2275 and miR395 were part of a different cluster.

**Figure 3:**
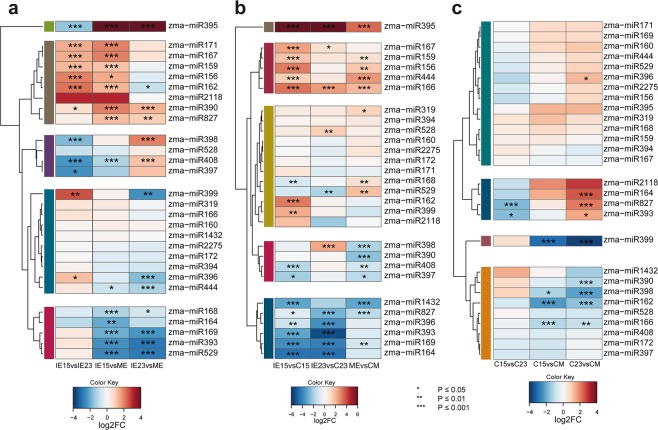
miRNA differential accumulation (DA) during callus induction from maize embryos collected at different developmental stages. (**a**) DA of miRNAs between zygotic embryos developmental stages (IE15, IE23 and ME). Comparisons were done for IE15 vs. IE23, IE15 vs. ME and IE23 vs. ME. (**b**) DA of miRNAs during the induction of Somatic Embryogenesis. Comparisons were done for IE15 vs. C15, IE23 vs. C23 and ME vs. CM. (**c**) DA of miRNAs in callus tissues obtained from developmentally distinct embryos at one month of induction. Comparisons were done for C15 vs. C23, C15 vs. CM and C23 vs. CM. Values of DA were expressed as the log2 Fold Change (log2FC) and significant values indicated as follows: *p < 0.05; **p < 0.01; ***p < 0.001.

Differential accumulation (DA) of miRNAs was examined through three different comparisons: (i) between embryos, (ii) callus induction between explants, and (iii) between calli. Statistically significant DA miRNAs were found between all embryo developmental stages, with greater differences for IE15 vs ME (Fig. 3a). miR156, miR159, miR162, miR167, miR171 and miR390 showed significantly higher levels in IE15, whereas miR168, miR169, miR393, miR444 and miR529 were preferentially accumulated in the ME and showed no differences between IE15 and IE23. Other miRNAs were characterized by unique patterns: miR395 significantly increased from IE15 to IE23 (2.3-fold change) but then dropped in ME (131-fold decrease compared to IE23); miR398 had a statistically significant increase in accumulation only for IE23. On the other hand, miR408 demonstrated the lowest abundance level in IE15, increased in IE23 and further decreased in ME (1.4-fold change). The miRNAs with lower accumulation in IE15 than IE23 are related to stress responses. Overall, these patterns may reflect the dynamic nature of miRNA levels during embryo development and, most importantly, its potential impact on the explant differential responses to dedifferentiation.

miR164, miR393, miR396, miR827 and miR1432 were highly accumulated in calli obtained from IE15 and IE23, showing significantly lower levels in the corresponding explants (Fig. 3b; bottom cluster). Concomitantly, miR156, miR159, miR166, miR167, miR395 and miR444 were more abundant in the zygotic embryo tissues (Fig. 3b; upper cluster). The difference was significant for all embryo developmental stages only for miR166 and miR395 (Fig. 3b; upper clusters). Besides the observed general responses, some miRNAs had a unique pattern for IE15, the explant with greatest embryogenic potential. Particularly, it was the only explant with significantly higher miR396 and miR399, and lower miR168 levels (Fig. 3b; clusters 3 and 5 from the top). In addition, miR398 and miR529 did not show significant DA during callus induction from IE15, while they were oppositely regulated in IE23 and ME inductions. These observations supported that readjustments for some miRNA families during dedifferentiation strongly depend on the explant developmental stage. Moreover, the starting miRNA level in the explant might have influenced their target regulation during callus induction. For example, miR156, miR166 and miR167 were significantly more abundant in the IE15 explant (Fig. 3a). While their abundance decreased in the induced callus for all explants (Fig. 3b), the final level of mRNA targets could partially depend on the miRNA initial amount.

Surprisingly, despite the contrasting morphological and embryogenic features between calli (Fig. 1g–q), significant differences in miRNA abundance were observed mostly for CM when compared to either C15 or C23. It displayed significantly higher levels of miR162, miR166, miR398 and miR399 than C15 and C23, and showed significantly lower levels of miR164, miR390, and miR396 than C23 (Fig. 3c). In addition, miR393 and miR827 showed significantly lower levels in C15 compared to C23 - a distinctive feature between these two calli types. A few miRNA families with known roles in developmental processes, miR162, miR166 and miR393, distinguished C15 and CM calli. Such differences might translate into amplified effects on several targets and their corresponding downstream activities. Furthermore, other sRNA populations might additionally impact the fate of the callus.

### miRNA target regulation in dedifferentiation, by explant developmental stage

miRNA regulation is exerted through the control of particular target abundances or translation. RNA-seq data was generated from the same samples used for sRNA analysis, and with these data, we inspected either predicted or experimentally confirmed miRNA targets (Figs S4–S6). As expected, significantly different abundances were found for several miRNA targets during the zygotic embryo development (Fig. S4). *SQUAMOSA BINDING PROTEIN ZmSBP2*, 3, 18 and 27 (miR156 targets), *b-HELIX-LOOP-HELIX ZmbHLH137* (miR162 target), *AUXIN RESPONSE FACTORS ZmARF16*, 18 and 30 (miR167 targets) were all up regulated at later developmental stages, inversely mirroring the miRNA accumulation (Fig. 3). On the other hand, *ZmSBP6*, targeted by miR529, as well as transcripts encoding histone-lysine N-methyltransferase member (*SUVH9*), *ZmSBP1* and *TRANSPORT INHIBITOR RESPONSE 1* (*TIR1*), targeted by miR393, showed significantly lower levels in the mature embryo (Fig. S3). The abundance of these targets negatively correlates with the higher miR393 and miR529 levels in the mature embryo (Fig. 3a).

Important differences in the *ZmSBP* family were also observed during dedifferentiation (Fig. S5). Significantly up-regulated transcripts were detected in C15 induction (*ZmSBP18*, 20, 22 and 29), whereas down-regulated SBP isoforms (*ZmSBP2*, 3, 8, 30, 32 and the a transcript orthologous to Arabidopsis *SPL18*) were more numerous during CM induction. On the other hand, *CUP-SHAPED COTYLEDON 2* (*ZmCUC2*), miR164 target, significantly decreased in C15, but not in C23 or CM callus induction. A set of *ZmARFs* targeted by miR167 showed differential patterns in C15 and C23 induction, such as *ZmARF22* (up-regulated for C15, but not for C23) and *ZmARF18* (down-regulated for C23, but not for C15). Other *ZmARFs* targeted by miR167 did not show a negative correlation with the miRNA during callus induction, suggesting additional levels of transcript abundance regulation or translational inhibition, rather than cleavage promoted by the miRNA.

Many targets of stress-related miRNAs showed an inverse pattern relative to their cognate miRNAs in calli, regardless of the developmental stage of the explant. For example, a group of *GROWTH REGULATOR FACTOR* (*GRF*) genes targeted by miR396, decreased in callus tissues. On the other hand, levels increased for transcripts of sulfate transporters and other sulfate metabolism-related proteins, targets of miR395. Curiously, in spite of the lack of significance in miRNA DA between C15 and C23, several miRNA targets showed significant DA in these tissues (Fig. S6). These included *ZmNAC113* (miR164 target) and *ZmGRF6* (miR396 target), more abundant in C23, as well as *Zm00001d025268* orthologous to *AUXIN SIGNALING F-BOX 3* (*AFB3*) and target of miR393, which was more abundant in C15. Hence, although the overall de-differentiation process implies similar miRNA readjustments for all explants, differential target accumulation patterns upon callus induction might result from both miRNA levels and transcriptional regulation of their target transcripts.

### Differential accumulation of tasiRNAs

Plant *TAS3* tasiRNAs function to regulate auxin responses during development; these so-called tasiARFs target members of the ARF transcription factor family^30^. Since callus induction occurs in response to high exogenous auxin levels, tasiRNAs may well function during the SE process^31^. In maize, nine *TAS3* loci (*TAS3a-i*) have been identified^32^. Eight of them produce mature tasiARFs that target transcripts of the ARF3 family (Table S4). The *TAS3a-c* loci produce two tasiARFs (1 and 2) each, yielding a total of eleven mature maize tasiARFs. However, tasiARF3b-1, tasiARF3b-2 and tasiARF3g are identical in sequence to tasiARF3c-1, tasiARF3c-2 and tasiARF3i, respectively, and were not differentiated in our analysis.

**Figure 4:**
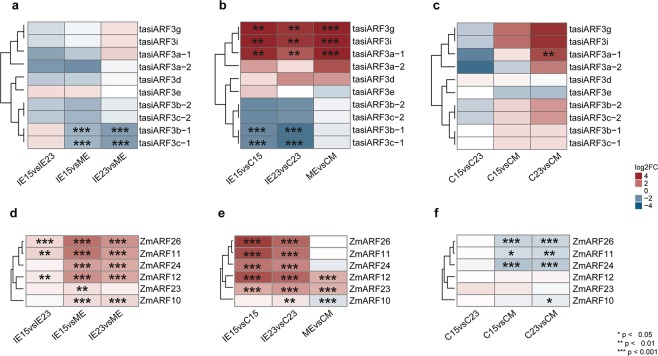
Regulation mediated by tasiARFs pathway during maize callus induction. (**a**) Differential accumulation (DA) of tasiARFs between zygotic embryos developmental stages (IE15, IE23 and ME). Comparisons were done for IE15 vs. IE23, IE15 vs. ME and IE23 vs. ME. (**b**) DA of tasiARFs during the induction of Somatic Embryogenesis. Comparisons were done for IE15 vs. C15, IE23 vs. C23 and ME vs. CM. (**c**) DA of tasiARFs in callus tissues obtained from developmentally distinct embryos at one month of induction. Comparisons were done for C15 vs. C23, C15 vs. CM and C23 vs. CM. (**d**–**f**) DA of Auxin Response Factors (ARFs) targets of tasiARFs. Comparisons were done as described for (**a**–**c**), respectively. Values of DA were expressed as the log2 Fold Change (log2FC) and significant values indicated as follows: *p < 0.05; **p < 0.01; ***p < 0.001.

The nine *TAS3* precursors were detected in IE15 and presented higher levels, except for *TAS3a*, *TAS3b*, *TAS3d* and *TAS3h*, which were more abundant in IE23 (Fig. S7a). *TAS3a*, *TAS3e*, *TAS3h*, and *TAS3i* precursors were only detected in zygotic embryos. Moreover, the *TAS3c* precursor exclusively appeared in IE15 and C15. All tasiARFs except tasiARF3f were detected in at least one tissue. Their overall abundance was higher in callus (Table S4). Particularly, tasiARF3b/c were the most abundant (Fig. S7b). They also showed significantly lower levels in IE15 and IE23, compared to ME (Fig. 4a). Two contrasting patterns of tasiARF abundances were observed during dedifferentiation. Mature tasiARF3a, tasiARF3d, tasiARF3g and tasiARF3i were more abundant in embryo tissues, while tasiARF3b/c were more abundant in calli (Fig. 4b). tasiARF up-regulation during dedifferentiation was statistically significant only for C15 and C23. Between the calli induced from different embryo explants statistically significant DA was observed for tasiARF3a-1 between C23 and CM (Fig. 4c).

To evaluate tasiARF-mediated regulation, we analyzed the sequences of 36 *ARF* genes reported for maize. Prior reports indicated five *ARFs* are targeted by tasiARFs^30,32^. Our analysis of the remaining 32 sequences identified an additional *ARF* possibly targeted by tasiARFs (*ZmARF10*). Based on the RNA-seq data, all TAS3-targeted *ARF* genes showed significant DA between the embryo developmental stages, particularly when compared to ME. The *ARF* patterns of accumulation inversely correlated with tasiARF3b/c abundances, showing higher accumulation in IE15 compared to IE23 and ME (Fig. 4d). During dedifferentiation, *ZmARF11*, *ZmARF24 and ZmARF26* transcripts were significantly reduced in C15 and C23, but not in CM (Fig. 4e). In addition, *ZmARF10* displayed up-regulation in CM. Consistent with these patterns, calli produced from IE15 and IE23 showed significantly lower levels of *ARF-*encoding transcripts (Fig. 4f), suggesting efficient regulation mediated by tasiARF3b/c particularly for these types of calli. In addition to tasiARF-regulated *ZmARF* family members, other *ARFs* were significantly decreased or increased during callus induction (Fig. S8). This is in agreement with a report on Arabidopsis SE, showing contrasting patterns for different *ARF* family members^33^.

### Validation of miRNA, tasiARF and target accumulation patterns by RT-qPCR

**Figure 5:**
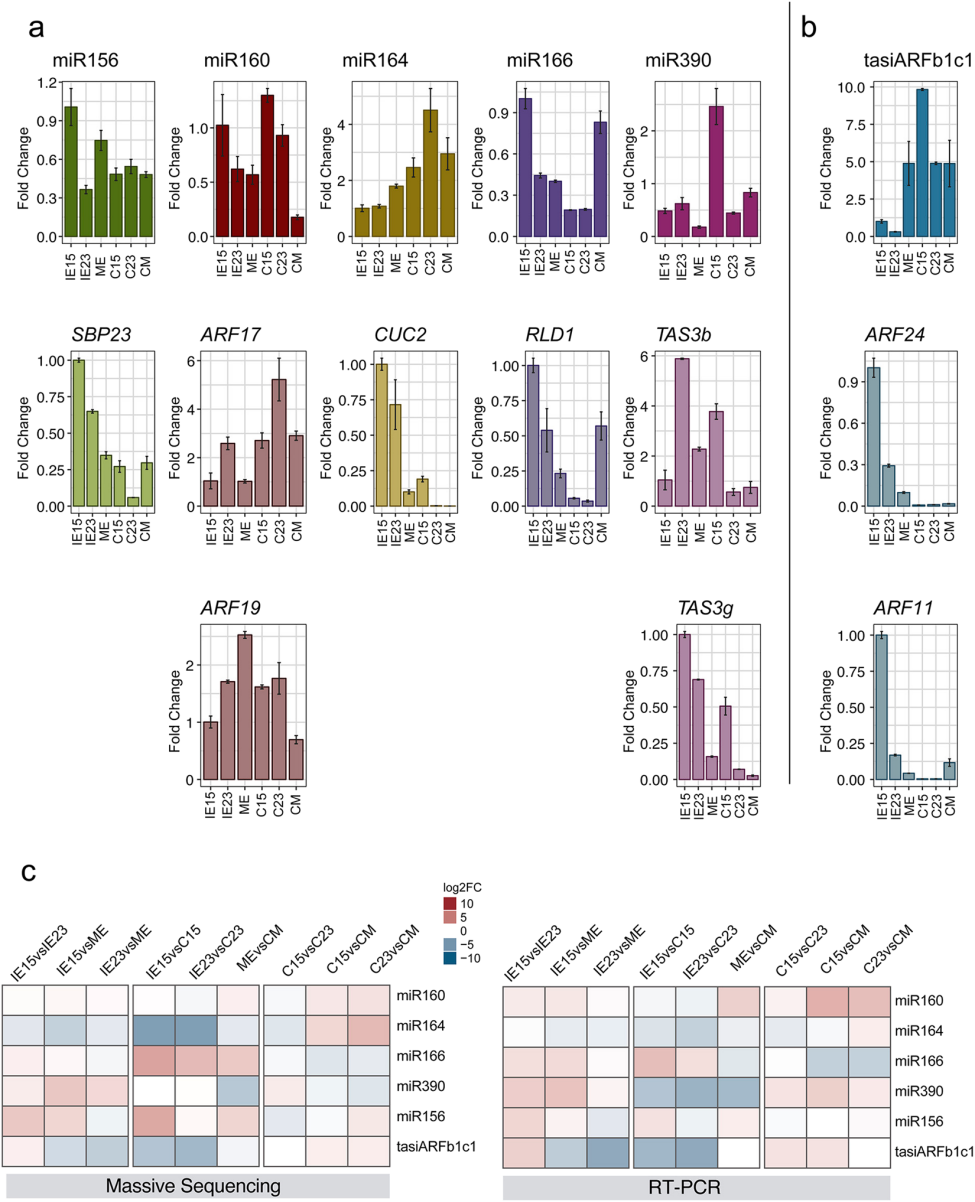
Relative abundances of selected sRNA and their corresponding targets during callus induction from zygotic embryos collected at distinct developmental stages. sRNA and mRNA abundances were analyzed by qRT-PCR in Tuxpeño VS-535 immature embryos (IE15, IE23) and mature embryos (ME) used as explants and the corresponding induced one-month callus tissues (C15, C23, CM). Accumulation of selected sRNAs (upper graphs, 1^st^ row) and their targets (lower graphs, 2^nd^ and 3^rd^ rows) are shown in similar bar color. (**a**) miRNAs and targets; (**b**) tasiARF and targets. Fold change represents abundances relative to IE15. sRNAs were normalized by U6 snRNA and targets by 18S rRNA internal controls. (**c**) Heat maps comparing differential accumulation of selected sRNAs by massive sequencing and qRT-PCR according to the value of log2FC.

To confirm the differential accumulation of miR156, miR160, miR164, miR166, miR390 and tasiARFb in maize embryos and induced calli, we used RT-qPCR and compared their patterns with the levels of some of their targets (Fig. 5). Most sRNAs showed similar abundances or trends to the high-throughput sequencing data, except miR390 (Fig. 5c). miR156, miR160 and miR166 displayed higher levels in the IE15 sample than in IE23 or ME (Fig. 5a). Upon callus induction, miR160 remained high for C15 and C23, but not for CM, miR164 increased in all calli, miR390 showed particularly high levels for C15 and miR166 abundance increased only for CM. Negative correlation was observed for miR160, miR164 and miR390 targets in C15 and C23, albeit differential patterns existed for targets of the same miRNA (miR160 and miR390). On the other hand, miR156 and miR166 reduction during callus induction was also accompanied by target decrease, except for CM, suggesting additional levels of regulation for these targets.

miR390 is required for the tasiARF biogenesis pathway for appropriate processing of *TAS3* precursors. This miRNA was more abundant in C15 than in other calli correlating with higher levels of tasiARFb1c1 (Fig. 5b). The TAS3b precursor accumulated in IE23 compared to the other explants correlating with low levels of miR390 and the tasiARF. Curiously, we were able to detect the *TAS3g* precursor but not the corresponding tasiARF (Table S4) suggesting some selectivity for processing by miR390-guided RISC. In addition, *ZmARF24* and *ZmARF11* showed strong negative correlation with tasiARFb accumulation upon callus induction (C15 and C23), consistent with the high-throughput sequencing data (Fig. 4).

### sRNAs mapped to transposable elements

**Figure 6:**
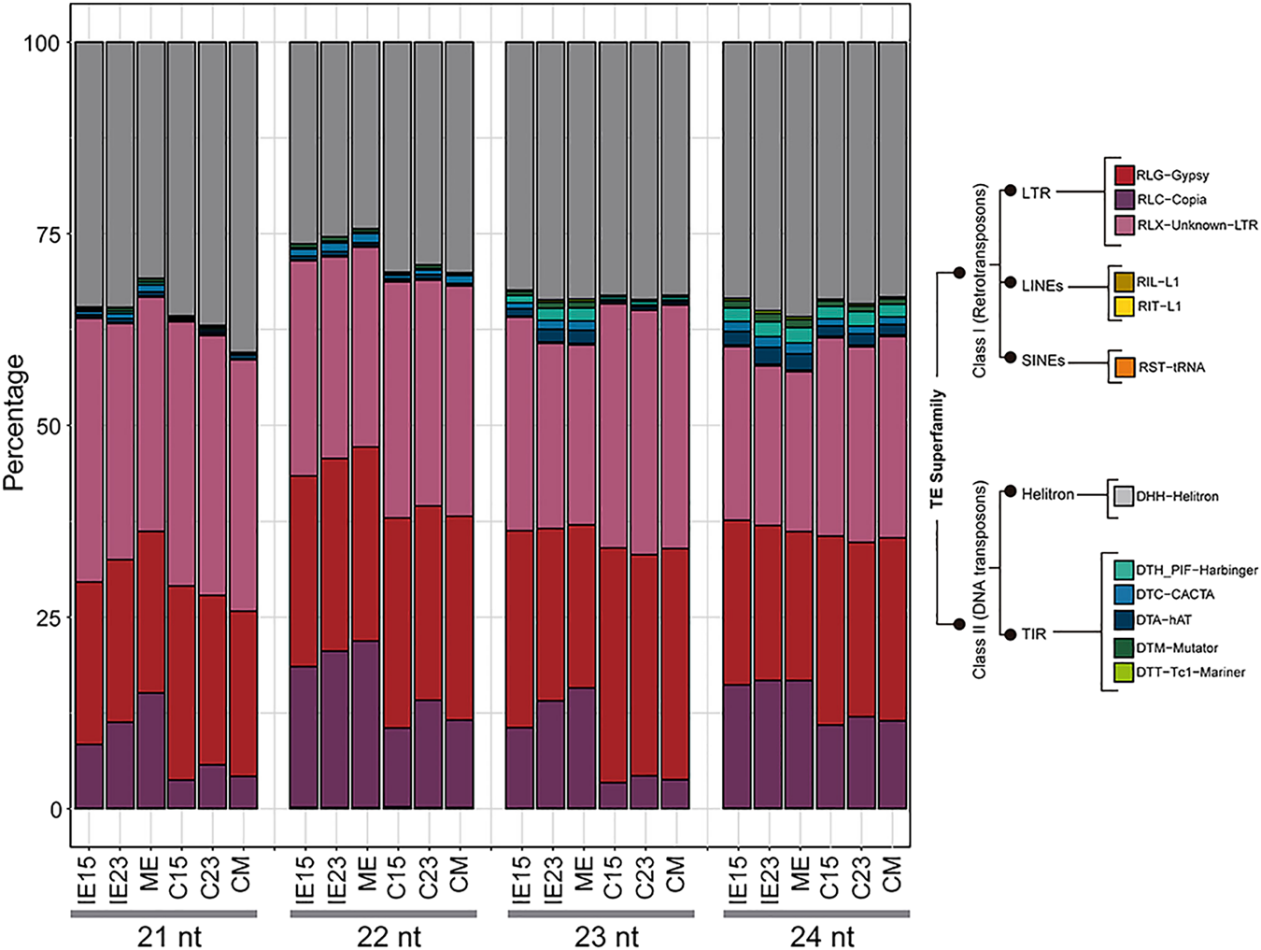
hc-siRNAs distribution in the different tissues. Relative abundance of normalized reads (represented as a proportion of total mapped reads) that mapped to major Transposable Element (TE) Superfamilies. **IE15:** Immature Embryos 15 days after pollination (DAP); **IE23:** Immature Embryos 23 DAP; **ME:** Mature Embryos; **C15**, **C23** and **CM:**Tissues one month after induction of Somatic Embryogenesis (SE) obtained from IE15, IE23, and ME, respectively.

The abundances of siRNAs matched to TEs and repeat regions in the maize genome were normalized according to the copy number of each TE superfamily (Table S5). Size analysis indicated that both 22- and 24-nt species were the most abundant for all tissues (Fig. S9). The 24-nt hc-siRNAs from all TEs sharply decreased in the induced callus, as compared to the corresponding embryo explant, whereas the 22-nt species decreased to a lesser extent (Fig. S9). Although the biogenesis of 22-mers mapping to TEs and repeats in maize is not well described^24^, we grouped them together with 24-nt hc-siRNAs, since they have a comparable genome distribution. LTR Class I retrotransposons (Copia, Gypsy and RLX-Unknown-LTR) accounted for more than 60% of the mapped sRNAs in all the tissues, followed by Helitron Class II DNA transposons (Figs 6 and S9). The proportion of hc-siRNAs from other TE superfamilies varied during callus induction. While the Copia-derived hc-siRNAs were reduced in the callus, hc-siRNAs from Gypsy and RLX-Unknown-LTRs were increased.

**Figure 7:**
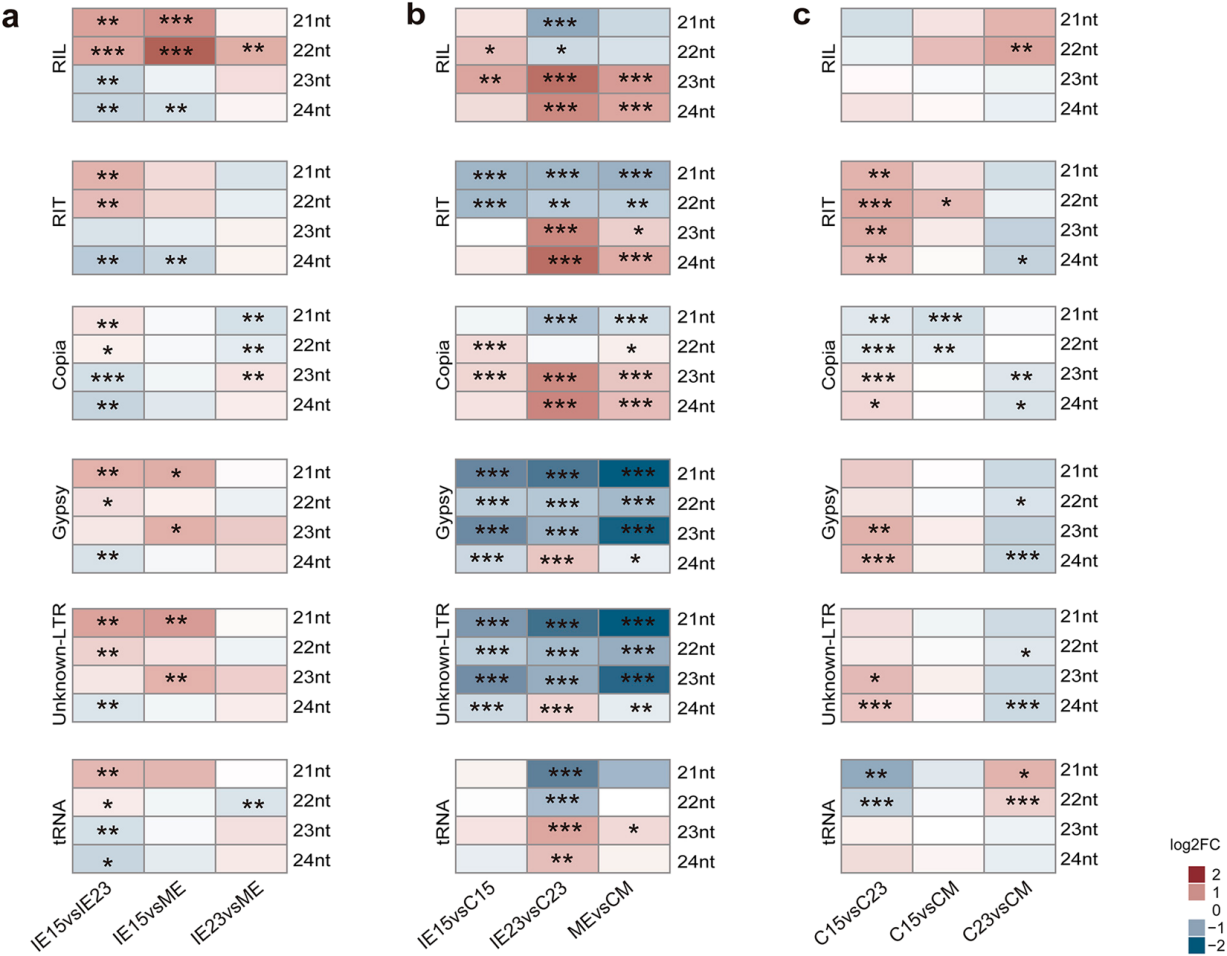
Differential accumulation (DA) of retrotransposon-matched hc-siRNAs during callus induction from maize embryos collected at different developmental stages. (**a**) DA of retrotransposon mapped hc-siRNAs between zygotic embryos developmental stages (IE15, IE23 and ME). Comparisons were done for IE15 vs. IE23, IE15 vs. ME and IE23 vs. ME. (**b**) DA of retrotransposon mapped hc-siRNAs during the induction of Somatic Embryogenesis. Comparisons were done for IE15 vs. C15, IE23 vs. C23 and ME vs. CM. (**c**) DA of retrotransposon mapped hc-siRNAs in callus tissues obtained from developmentally distinct embryos at one month of induction. Comparisons were done for C15 vs. C23, C15 vs. CM and C23 vs. CM. Values of DA were expressed as the log2 Fold Change (log2FC) and significant values indicated as follows: *p < 0.05; **p < 0.01; ***p < 0.001.

One intriguing change upon dedifferentiation was a reduction in 23/24-nt, but not in 21/22-nt sRNA abundances. Significant DA was found to greater extent between IE15 and IE23 than between IE23 and ME (Fig. 7). For example, in IE15, the 21- or 22-nt Class I Retrotransposon-mapped sRNAs were up-regulated, while the 23- or 24-nt sRNAs were down-regulated. Upon dedifferentiation (embryo vs. callus), 24-nt hc-siRNAs derived from Gypsy and LTR-unknown TE showed opposite patterns for IE15 and IE23. Comparable behavior was found for the Helitron-mapped sRNAs, representing the second most abundant order of TEs (Fig. S10). sRNAs mapping to Copia TEs were more abundant in embryo tissues, except for the 21-nt class. Significant DA was even observed when comparing the calli derived from IE15 and IE23 (Fig. 7c). In particular, Copia, Gypsy and Unknown-LTR TEs, as well as Helitron-derived 23 and 24 nt sRNAs were increased in C15 compared to C23, while Copia-derived 21–22 nt species were decreased. Although a reliable interpretation of such changes is not possible at this moment due to limited work on hc-siRNA pathways in maize, they may impact the regulation of SE.

## Discussion

Our findings support a role of differential sRNA (miRNA, tasiRNA and hc-siRNA)-mediated regulation during maize callus induction depending on the explant developmental stage. Previous studies demonstrated that sRNA abundances change during maize zygotic embryo development and upon callus induction from 15 DAP immature embryos^11,13,34^. However, the impact of sRNA-target balance present in the explants, during dedifferentiation and in the induced callus was not explored. Zygotic embryos at 23 DAP strongly decreased in the callus embryogenic potential and mature embryos were unable to properly establish dedifferentiation under the 2,4-D stimulus. Similitudes were observed between C15 and C23, including the presence of procambial and parenchymatic cells, possibly as a response to the auxin and cytokinin stimuli^35^. The abundant presence of tracheal elements suggests they have a role in water transport across tissues, whereas the parenchymatic cells could function as supporting tissue. Nevertheless, IE23 generated mostly non-embryogenic callus and showed low proliferation rates accompanied by higher oxidation. Such strikingly different phenotypes reflect the inability to achieve the genetic reprogramming required for embryogenic competence in the explant.

Consistent with the embryo distinct morphological features, the accumulation of many development-related miRNAs^36,37^ decreased at stages beyond 15 DAP, whereas stress- and nutrient transport-related miRNAs^38^ significantly increased. In addition, dedifferentiation from IE15 resulted in more significant miRNA accumulation switches than for the other explants. Calli induced from IE23, displayed increases for most stress-related miRNAs, but did not show significant DA for development-related miRNAs, except for miR166. Actually, miR166 and miR395 showed the most striking reduction in calli for all explants and might represent a general response to the de-differentiation stimulus. Similar miRNA patterns observed during dedifferentiation could be interpreted as abundance adjustments dictated by the imposed stress and hormone concentration^12^. Ultimately, transcription factors targeted by miRNAs would regulate the precise response to external stimuli^22,37^. Consistently, significant differences for miRNA targets were evident between calli generated from the distinct explants and might better correlate with their distinct embryogenic potential.

tasiARFs were recently studied in *Dimocarpus longan* plant regeneration through SE^31^ and, in a previous analysis, we detected a transient increase for this sRNA group during VS-535 dedifferentiation^13^. In maize, all tasiARFs are derived from TAS3 precursors^30^. We observed that precursor abundances decreased upon dedifferentiation, whereas tasiARF3b/c significantly increased, consistent with either more efficient precursor processing or stabilization of these tasiARFs. Importantly, miR390 levels correlated with the processing of the *TAS3b* precursor and the accumulation of tasiARFb/c during callus induction from IE15, suggesting specifically guided tasiRNA biogenesis within the process. The tasiARF stimulation and corresponding *ARF* down-regulation were particularly evident for calli with higher embryogenic potential (C15 and C23). This tasiARF-mediated regulation in callus induction nicely correlates with the observed decrease of miR166, a downstream ARF-regulated target^32^.

**Figure 8:**
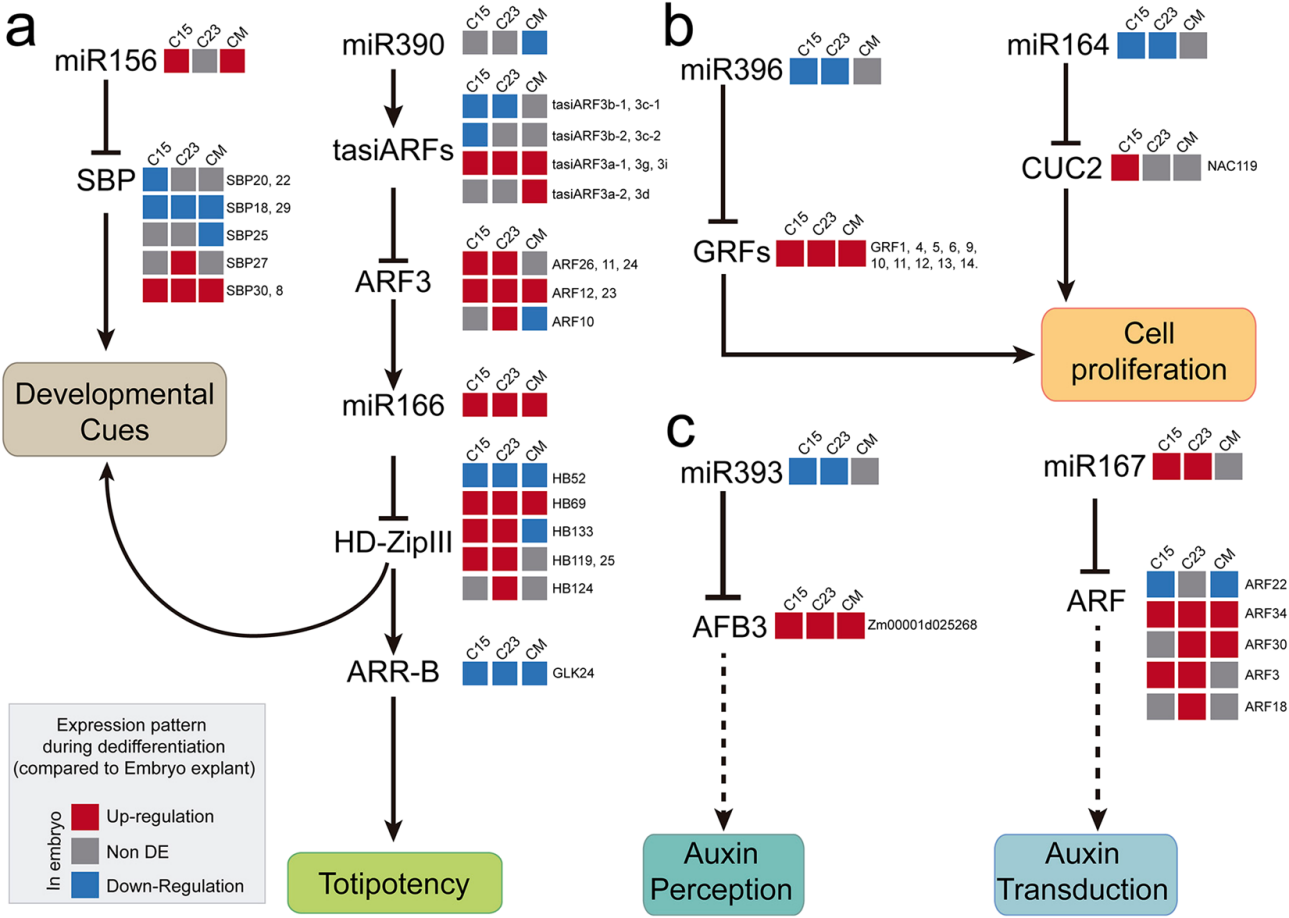
Model for miRNA- and tasiARF-mediated regulation during maize callus induction. (**a**) miRNA and tasiARF nodes shaping developmental transitions and cell totipotency maintenance. (**b**) miRNA nodes impacting cell proliferation. (**c**) miRNA nodes for auxin signaling. The cellular processes affected by miRNA and tasiARF regulation are depicted according to studies developed mainly in *Arabidopsis thaliana* and maize^39,64^. Arrows indicate positive and T-shape negative regulation. The broken arrows represent suggestive or speculative relationships for maize. The expression pattern of sRNAs and their targets during C15, C23 and CM induction is based on the results provided by this work and is represented colored squares as explained in the box. Gene name designations close to the squares refer to different maize transcripts corresponding to the same family in the model (*ZmSBP20*, 22), whereas those within the pathways are referenced to *Arabidopsis thaliana* orthologous genes (i.e. *AFB3*).

In 2011, Rubio-Somoza and Weigel^39^ described miRNA- and tasiRNA-mediated regulation by particular nodes operating for proper developmental programs and environmental responses in the model plant *Arabidopsis thaliana*. In addition, a recent review described the relevance of miRNA nodes function in economically relevant crops, such as rice and maize^38^. Taking this into account, we analyzed the operational status of some of these nodes and their intersection with the cellular processes taking place during dedifferentiation in maize SE (Fig. 8). Based on our data, miR156, miR166 and tasiARFs likely function somehow differently in IE15 to regulate the developmental switch taking place during dedifferentiation, as well as the acquisition of cellular totipotency (Fig. 8a). miR156 regulates several SBP transcripts known to modulate developmental responses during zygotic and somatic embryo development^22,23,40^. C15 presented higher levels of *ZmSBP20* and *ZmSBP22*, in addition to *ZmSBP18* and *ZmSBP19* increased for all calli, representing more targets with negative correlation to miR156 levels during dedifferentiation. miR156 and miR166 appear connected in the same pathway to achieve regenerative responses in *Arabidopsis thaliana* SAM^41^. On the other hand, tasiARF-mediated regulation in response to auxin results in *HD-ZipIII/RLD1* transcription factor regulation of shape - not only morphogenetic patterns, but also totipotent cell niches during *in vitro* regenerative responses^41,42^. Dedifferentiation from ME did not show a significant increase of tasiARFs as compared to IE15 and IE23, possibly contributing to its non-embryogenic fate. It is important to highlight that some HD-ZIPIII transcription factors such as *ZHD25*, *ZHD58*, *ZHD119* and *ZHD133* displayed differential accumulation patterns for calli induced from the different explants. This could be relevant for the ultimate dedifferentiation status achieved by the tissue.

In a different miRNA node (Fig. 8b), miR164 and miR396 both impact the regulation of cell proliferation^39^. Interestingly, these miRNAs increase during callus induction from IE15 and C15 accompanied by significant *CUC2* down-regulation. This could reflect the initial miR164 and *CUC2* levels found in the embryo explant, since it is known that this transcript has very restricted timing and cell type expression patterns during the zygotic embryo development^43^. Overall, during callus induction, the miR164/miR396 node might act as negative input on cell proliferation during dedifferentiation. This would be a premise for further successful proliferation of callus tissues^5^, which was impaired for explants different from IE15. Finally, the miR167/miR393 node (Fig. 8c) functions in plant auxin signaling, particularly, but not exclusively, for root auxin perception and transduction^39^. Supporting the high relevance of this hormone signaling in SE, we found a significant increase in miR393 and decrease in miR167 during dedifferentiation from IE15 and IE23. However, while *Zm00001d025268* (*AFB3* in Arabidopsis) targeted by miR393 decreased in all calli, an increase in *ZmARF22* (*ARF6* in Arabidopsis) targeted by miR167 was not observed for C23. Additionally, several ARF family members simultaneously decreased in C23. According to their role in auxin perception and transduction pathways^44^, we could infer that in the early induced calli, the excess of exogenous auxin (2,4-D) causes a decrease in auxin perception, but an increase in the pathway of signal transduction, which probably facilitates the appropriate reprogramming according to the stimulus. Particularly for this node, there were important differences for callus induction from IE23 and ME explants. This could explain in part the strikingly different phenotype of calli generated from ME, such as the massive root growth observed for these tissues.

hc-siRNAs comprise the least-explored sRNA group in the process of plant SE. However, their contribution to DNA methylation, histone modifications and chromatin remodeling might have great impact on genetic reprogramming under high hormone concentrations and stress conditions^5^. We previously evidenced shifts in different size classes of hc-siRNAs for VS-535 maize embryogenic calli induced from 15 DAP immature embryos^13^. Here we confirmed that the reduction in 23/24-nt hc-siRNA abundances characterizes all induced calli, regardless of the embryo explant developmental stage. However, the reduction was more significant for calli induced from IE23 and ME explants, and consequently, the calli induced from IE15 displayed significantly higher levels of these hc-siRNAs. Strikingly, major differences were found between C15 and C23 for hc-siRNAs mapping to both Class I retrotransposon and Class II DNA TEs, contrary to what was observed for miRNAs and tasiARFs. Such differences raise the possibility of differential TE regulation in C23, which might additionally contribute to its poor embryogenic potential or ability to respond to the stress imposed during dedifferentiation.

Overall, we have shown that several sRNA regulatory nodes operate differentially in 15 DAP maize immature embryos as compared to later developmental stages. According to their role in mRNA target regulation, we propose that they are particularly relevant for the required explant plasticity during dedifferentiation, further achievement of embryogenic potential and proliferation, as well as the proper management of auxin signaling and stress responses.

## Material and Methods

### Plant material and pollinations

All experiments were performed with the Mexican cultivar VS-535, the Tuxpeño landrace of maize (*Zea mays* L.). Plants were grown in a 30 kg sack with a commercial soil mixture (Sunshine) in a greenhouse at daylight and temperatures between 25–30 °C. Plants were watered three times a week. Flowering began at approximately four months of growth and ears were pollinated manually^45^. Immature embryos were collected at 15 and 23 DAP, IE15 and IE23 respectively, from the middle part of the ears, taking care to select similar sizes for each stage. Mature embryos (ME) were collected at 45 days after pollination. Collected embryos were divided in different batches for induction of somatic embryogenesis, morphological characterization, and RNA extractions.

### Callus induction from zygotic embryos

Callus induction was performed in the dark on N6 initiation medium^46^ including 2 mg L^−1^ of 2,4-D^47^. Induction of callus masses occurred after two weeks. The induced embryos were subcultured, after removing dead parts of the initial explant, on N6 maintenance medium, which included 2 mg L^−1^ of 2,4-D and 0.1 mg L^−1^ of kinetin. After two additional weeks (one month after induction), the status of induced callus was registered, and the tissue was sampled for morphological characterization (15–20 embryos and 20–25 callus portions induced from independent embryos, for each developmental stage) and RNA extractions (one gram per replicate for each embryo and callus developmental stage separated in 0.2 g pools for independent RNA extractions). The tissues obtained from IE15, IE23 and ME at one month of induction of SE were named as a C15, C23 and CM, respectively.

### Evaluation of embryogenic potential

The evaluation of callus embryogenic potential was performed according to the method reported by Gonzalez *et al*.^7^ at one month after induction. The Line Index (LI) was determined according to the callus phenotype as follows: 0 = without response; 1 = massive root formation; 2 = watery callus; 3 = compact, organogenic callus; 4 = low embryogenic structures; 5 = friable, low embryogenic structures; 6 = friable, highly embryogenic structures (embryogenic callus type II). Categories between 0 and 3 are non-embryogenic responses, whereas those between 4 and 6 are embryogenic responses. An explant with average LI above 3 was considered as “embryogenic”. The Induction Capacity (IC) was calculated by measuring the callus frequency in each category at one month after callus induction. Statistical analysis was performed in XLSTAT 2017 for the analysis of variance (ANOVA), and using Tukey´s test for group comparisons (Table S1).

### Histological analysis

Embryos and callus tissues were fixed, sectioned and stained as reported previously^48^. Embryo sections were performed by 12 μm longitudinal cuts and stained using the Safranin O/Fast Green Stain technique.

### RNA extraction and processing

Two biological replicates were used for each embryo and callus sample. RNA was isolated and fractionated by size using the Quick-RNA^TM^ MiniPrep system (Zymo Research). This method allowed simultaneous separation of large (>200 nt) and small (17 to 200 nt) from each sample^47^. The quality of each RNA fraction was tested by 1% agarose-gel electrophoresis, as well as with the Agilent 2100 Bioanalyzer. The RNA Integrity Number (RIN) for large RNAs ranged between 6.7 and 9.4 for all samples. For RT-qPCR validation experiments reverse transcription (RT) was performed using either an oligo (dT) primer (large RNAs) or stem-loop primer (miRNA and tasiARF) and the ImProm-II™ reverse transcription system (Promega, USA) as described^47^. Primer sequences used in this work are available in Supplementary methods. Reactions of qPCR were performed in triplicate with Maxima SYBR Green/ROX Master mix (ThermoFischer) in a 7500 Real-time PCR System (Applied Biosystems, USA). Abundances were expressed as “fold change” using the 2^−∆∆Ct^ method^49^, considering the IE15 sample as reference and rRNA 18S or U6 snRNA as internal housekeeping controls, for large and small RNAs respectively.

### Library sequencing and processing

Twelve sRNA and RNA-seq libraries (IE15, IE23, ME, C15, C23, CM; explained in Results), each with two biological replicates, were constructed using the small RNA (17–200 nt) and the large RNA (>200 nt) fractions, respectively. Sequencing was performed with Illumina NextSeq-2000 analyzer for sRNAs using single-end reads, and Illumina HiSeq 2000 analyzer for RNA using paired-end reads. Sequencing and library construction were performed by Unidad Universitaria de Secuenciación Masiva de DNA (UUSMD, UNAM-IBT, Mexico). RNA and sRNAs reads were first trimmed to remove adapters and filtered for quality above PHRED score 20 using Trimmomatic 0.36^50^. Quality visualization was performed using Fastqc (http://www.bioinformatics.babraham.ac.uk/projects/fastqc/).

### sRNA and RNA-seq read mapping and analysis

Filtered RNA-seq and sRNA reads were mapped with Bowtie2^51^ against the maize B73 Reference Genome (AGPv4.37) obtained from Gramene^52^ (ftp://ftp.gramene.org/pub/gramene/release-56/fasta/zea_mays/dna/). The paired-end format with default parameters (allowing a maximum of two mismatches) was used for RNA-seq reads and the single-end format with -n assigned as “1” to allow one mismatch for sRNA reads (Table S2). Sequences mapping to tRNA, rRNA, snRNA, and snoRNA loci were not included in sRNA analyses. Alignment BAM final files were converted to Fastq files with bamtofastq from Bedtools v2.26.0 toolset^53^. To control the read length (18 to 27 nt) and to generate tag-count files (i.e. sequence plus abundance) we used a published preprocessing script^54^. Reads in each library were adjusted to Reads Per Ten Million (RPTM). Details about miRNA, tasiRNA and hcsiRNA sequence identification are available in Supplementary methods. Transcript abundance quantification was performed using RSEM v1.2.31^55^ and the genomic features of maize genome (AGPv4.37) were retrieved from Ensembl gtf and gff3 files^56^.

### miRNA target identification

Prediction was performed using two bioinformatics platforms: (1) the Target Prediction tool from the Next-Gen Sequence Database^57^ using standard parameters; (2) the psRNATarget online platform^58^ using the V2 Schema. Besides these tools, we used public PARE libraries from different maize tissues, available from the Next-Gen Sequence Database (GEO Accession numbers: GSM1262608, GSM1262609, GSM1262610, GSM1262611, GSM1262612, SRX300975, SRX300976, SRX300977, SRX300978) to obtain experimentally validated miRNA targets by the tool comPARE (PARE Validated miRNA Targets)^57^.

### Differential accumulation analysis

Differential accumulation analyses were performed using the R programming language^59^ following the standard protocol from the DESeq2 package^60^, with the p-adj values for the log2FC significance (p-adj < 0.05). All graphs were developed using the R packages gplot^61^, ggplot2^62^ and ComplexHeatmap^63^.

## Supplementary information

Supplementary information

Dataset 1

Dataset 2

Dataset 3

Dataset 4

Dataset 5

## Data Availability

The sRNA and RNA-seq data sets generated in this study were deposited in the National Center for Biotechnology Information (NCBI) Gene Expression Omnibus (GEO) under the accession number GSE120438.
