# Supplementary material for: The explant developmental stage profoundly impacts small RNA-mediated regulation at the dedifferentiation step of maize somatic embryogenesis
Source: Sci Rep. 2019 Oct 10;9:14511. doi: 10.1038/s41598-019-50962-y (PMC6786999; doi:10.1038/s41598-019-50962-y)
Supplement: Supplementary file 1 — Supplementary information [file 41598_2019_50962_MOESM1_ESM.pdf]

Supplementary Information

The explant developmental stage profoundly impacts small RNA-mediated regulation at the dedifferentiation step of maize somatic embryogenesis

Vasti T. Juárez-González[1], Brenda A. López-Ruiz[1], Patricia Baldrich[2], Eduardo Luján-Soto[1], Blake C. Meyers[2,3], Tzvetanka D. Dinkova[1*]

Affiliations:

[1] Departamento de Bioquímica, Facultad de Química, Universidad Nacional Autónoma de México, 04510 CDMX, México

[2] Donald Danforth Plant Science Center, 975 North Warson Road, St. Louis, MO 63132, USA

[3] Division of Plant Sciences, University of Missouri, Columbia, Missouri 65211, USA

Corresponding author:

Tzvetanka D. Dinkova

Departamento de Bioquímica

Facultad de Química

Universidad Nacional Autónoma de México

04510 CDMX

cesy@unam.mx

Tel: + 52 55 56225277

Supplementary Figures

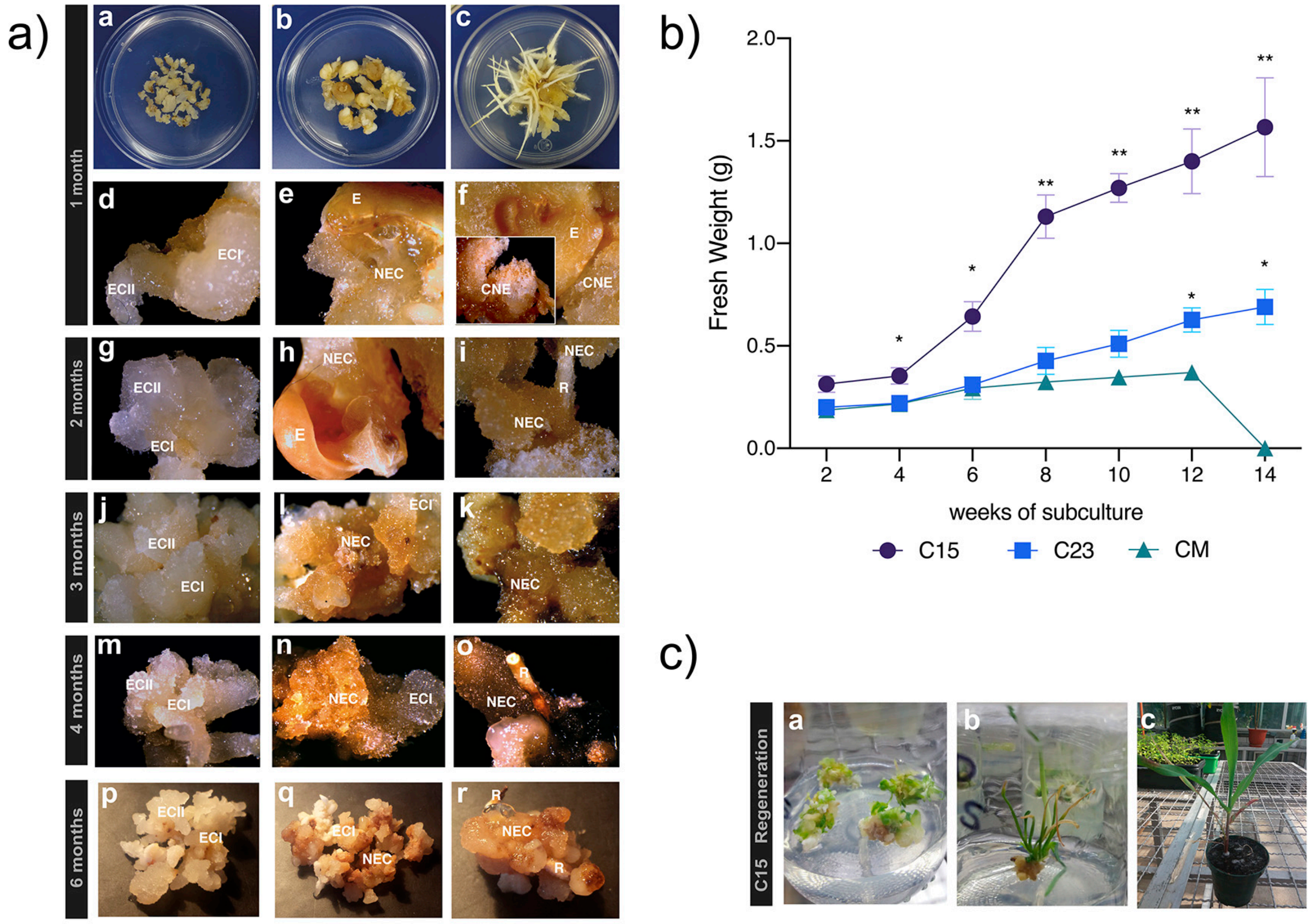

Fig. S1 Differential phenotypes of calli induced from maize VS-535 IE15, IE23 and ME. a) Follow-up at different sublculture times (1-6 months) for C15 (left panels), C23 (middle panels) and CM (right panels). ECI: embryogenic callus type I; ECII: embryogenic callus type II; NEC: non-embryogenic callus; R: root. **b)** Growth profiles of C15, C23 and CM for 14 weeks upon induction. For each time point, fresh weight was measured in three different samples of callus. The mean was plotted with bars indicating SEM. Data were analyzed using a Two-way ANOVA with Tukey's multiple comparison test. (*) $p < 0.05$; (**) $p < 0.01$. **c)** Plant regeneration from C15 at one year of subculture. a: 2 weeks on light and hormone depletion; b: 2 weeks on light hormone depletion + 2 weeks on MS medium; c: 2 weeks on light hormone depletion + 2 weeks on MS medium + 4 weeks on soil. C23 were not able to regenerate plants and CM were dead tissues upon 4-6 months of subculture.

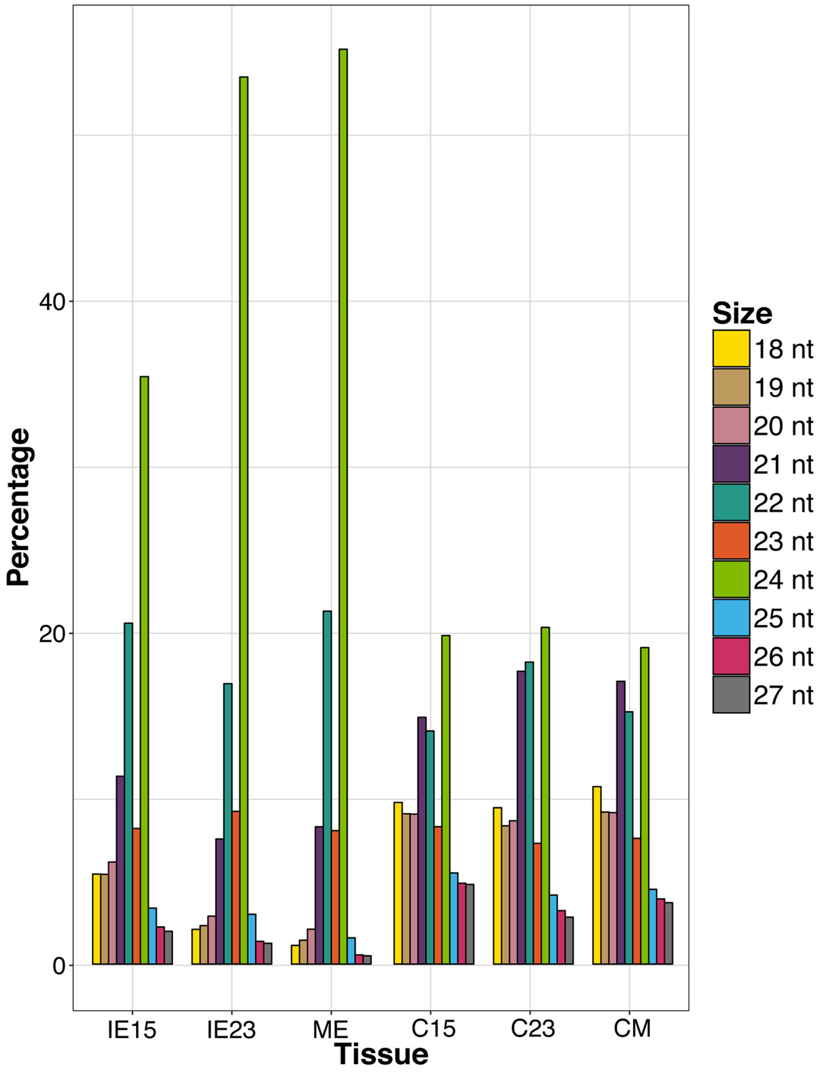

Fig. S2 sRNA library size distribution in the different maize embryos and induced callus tissues. Size distribution is shown by percentage of normalized reads per 10 million (RPTM) for each sRNA size from the total (18-27 nt). Mean values of the two biological replicates were used. IE15: Immature Embryos 15 days after pollination; IE23: Immature Embryos 23 days after pollination; ME: Mature Embryos; C15, C23 and CM: Callus tissues at one month after induction from IE15, IE23, and ME, respectively.

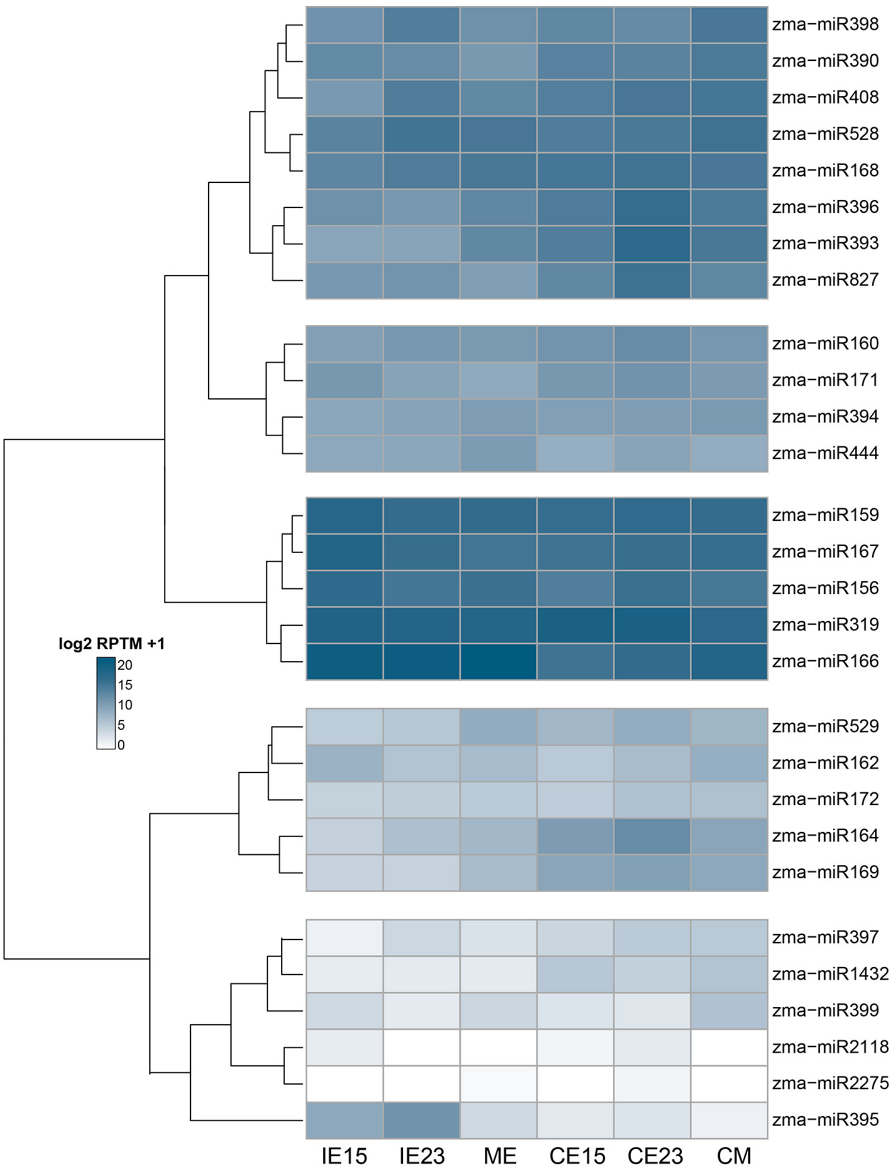

Fig. S3 Clustering of miRNA families by normalized abundance. Expression values are shown as a log2 normalized abundance (Reads per ten million; RPTM). Immature Embryo at 15 days after pollination; IE15. Immature Embryo at 23 days after pollination; IE23. Mature Embryo; ME. Callus from IE15, IE23 and ME at one month of induction (C15, C23, and CM, respectively).

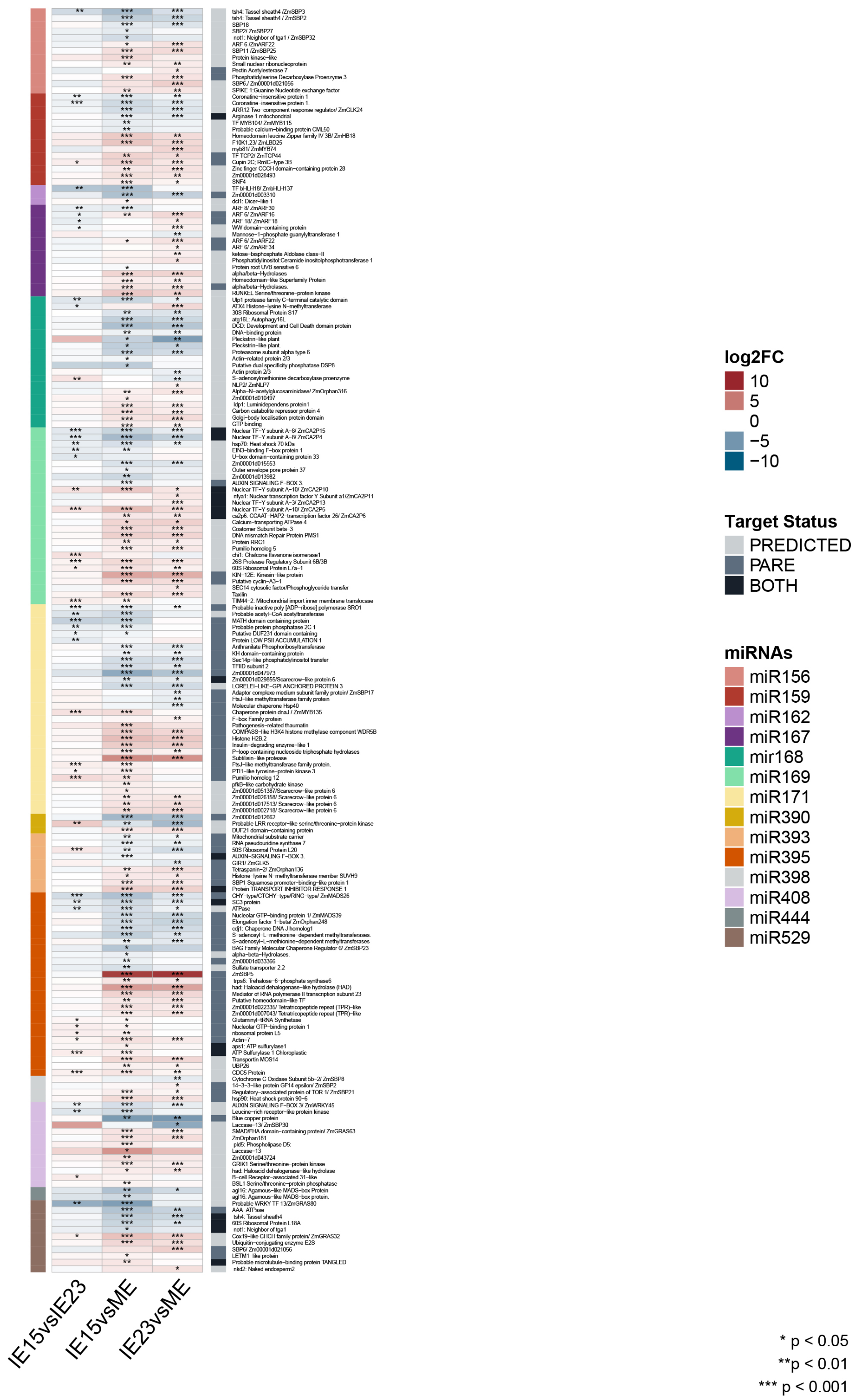

Fig. S4 Differential accumulation of miRNA targets during Zygotic Embryo development. Comparisons were done for IE15 vs. IE23, IE15 vs. ME and IE23 vs. ME. Values of DE were expressed as the log2 Fold Change (log2FC) and significant values indicated as follows: (*) $p<0.05$; (**) $p<0.01$; (***) $p<0.001$. Targets of each miRNA are designated with the left multiple color bar. The status of each target was referred to as identified by bioinformatics tools "Predicted", experimentally proved "PARE" or both methods "Both" according to the right grayscale bar.

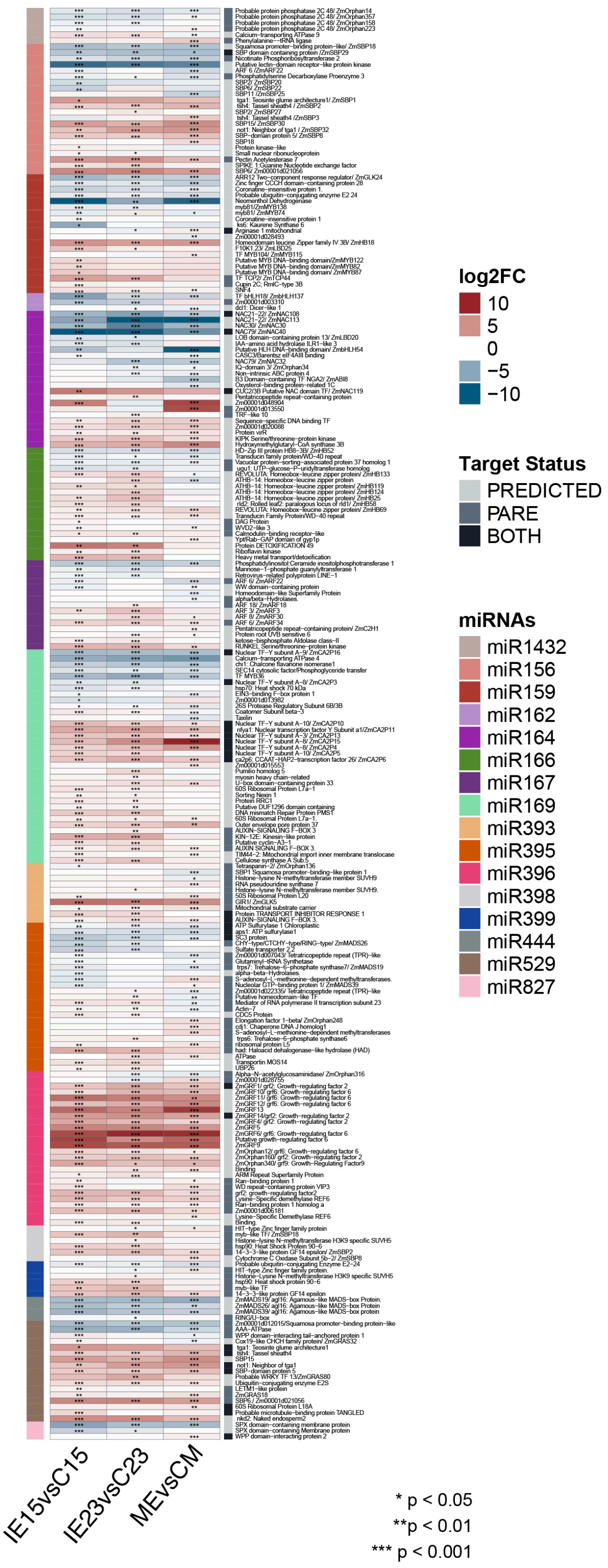

Fig. S5 Differential accumulation of miRNA targets during callus induction. Comparisons were done for IE15 vs. C15, IE23 vs. C23 and ME vs. CM. Values of DE were expressed as the log2 Fold Change (log2FC) and significant values indicated as follows: (*) $p<0.05$; (**) $p<0.01$; (***) $p<0.001$. Targets of each miRNA are designated with the left multiple color bar. The status of each target was referred to as identified by bioinformatics tools "Predicted", experimentally proved "PARE" or both methods "Both" according to the right grayscale bar.

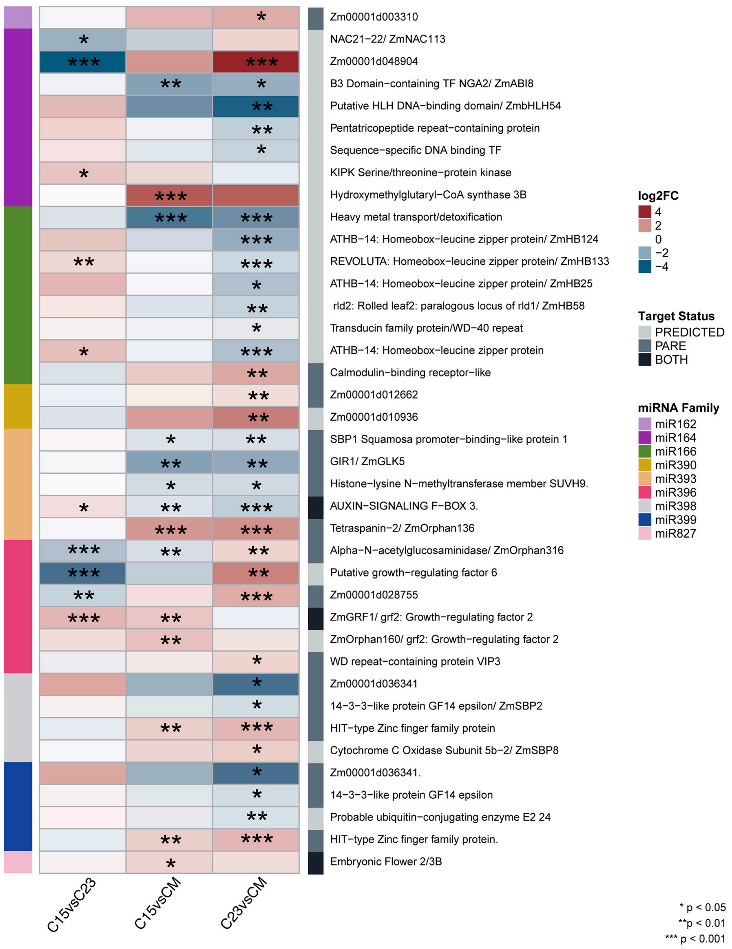

Fig. S6 Differential accumulation of miRNA targets in callus tissues. Comparisons were done at one month of induction from developmentally distinct embryos for C15 vs. C23, C15 vs. CM and C23 vs. CM. Values of DE were expressed as the log2 Fold Change (log2FC) and significant values indicated as follows: (*) $p<0.05$; (**) $p<0.01$; (***) $p<0.001$. Targets of each miRNA are designated with the left multiple color bar. The status of each target was referred to as identified by bioinformatics tools "Predicted", experimentally proved "PARE" or both methods "Both" according to the right grayscale bar.

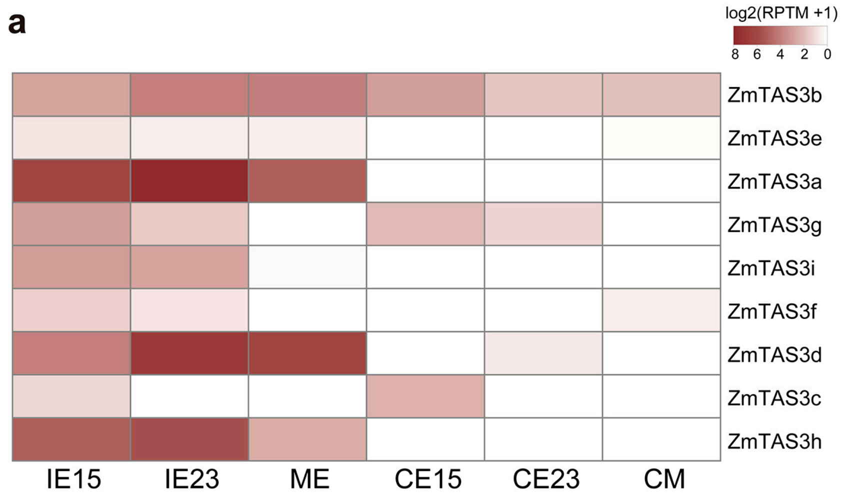

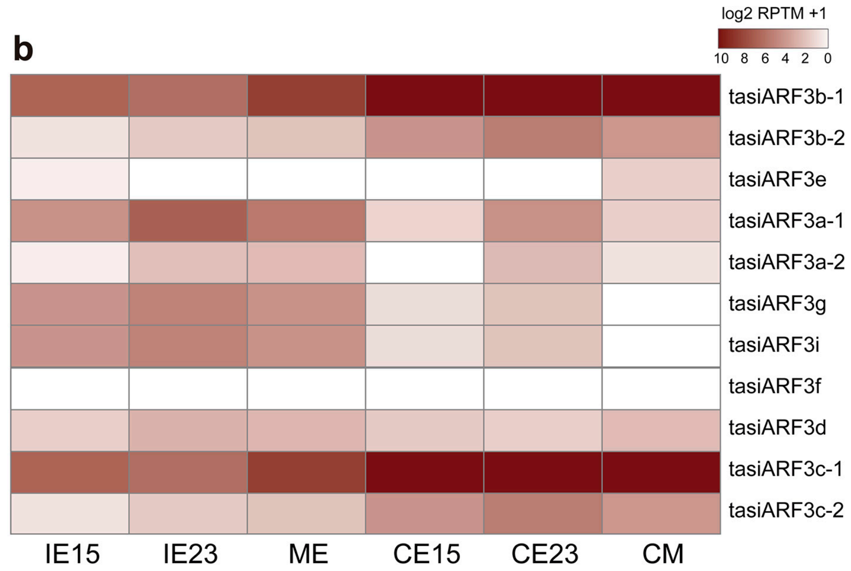

Fig. S7 TAS3 genes and tasiARFs normalized abundance. (a) TAS3 precursor abundances. (b) mature tasiARF abundances. Expression values are shown as a log2 normalized abundance (Reads per ten million; RPTM). Immature Embryo at 15 days after pollination; IE15. Immature Embryo at 23 days after pollination; IE23. Mature Embryo; ME. Callus from IE15, IE23 and ME at one month of induction (C15, C23, and CM, respectively).

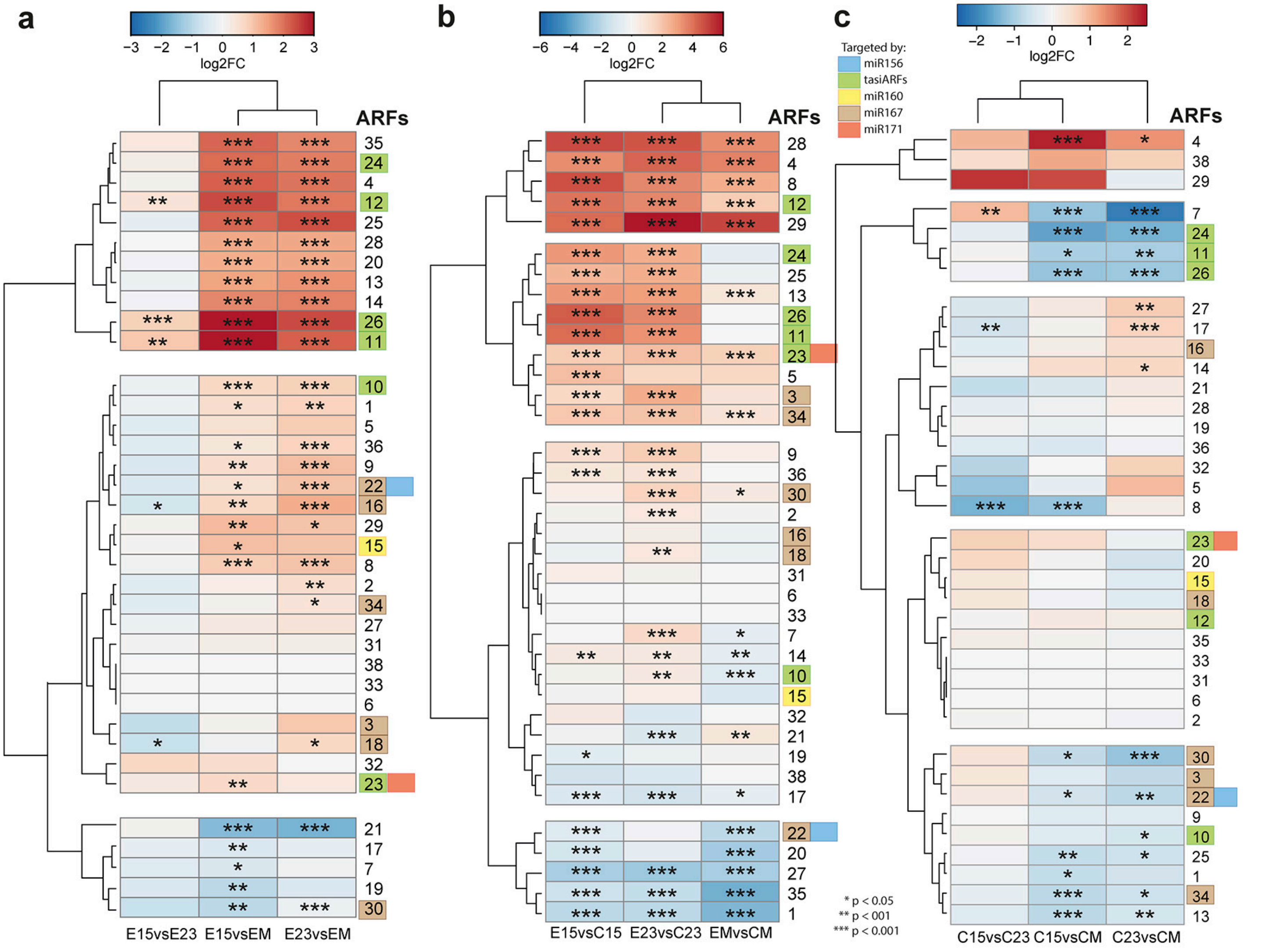

Fig. S8 Maize *AUXIN RESPONSE FACTOR* genes (*ZmARFs*) differential accumulation in callus induction from maize embryos collected at different developmental stages. (a) DE of *ZmARFs* between zygotic embryo developmental stages (IE15, IE23 and ME). Comparisons were done for IE15 vs. IE23, IE15 vs. ME and IE23 vs. ME. (b) DE of *ZmARFs* during the induction of Somatic Embryogenesis. Comparisons were done for IE15 vs. C15, IE23 vs. C23 and ME vs. CM. (c) DE of *ZmARFs* in callus tissues obtained from developmentally distinct embryos at one month of induction. Comparisons were done for C15 vs. C23, C15 vs. CM and C23 vs. CM. Values of DE were expressed as the log2 Fold Change (log2FC) and significant values indicated as follows: (*) p<0.05; (**) p<0.01; (***) p<0.001. Colored squares represent the sRNA targeting particular *ZmARF* as depicted on the figure. The numbers refers to the maize ARF nomenclature.

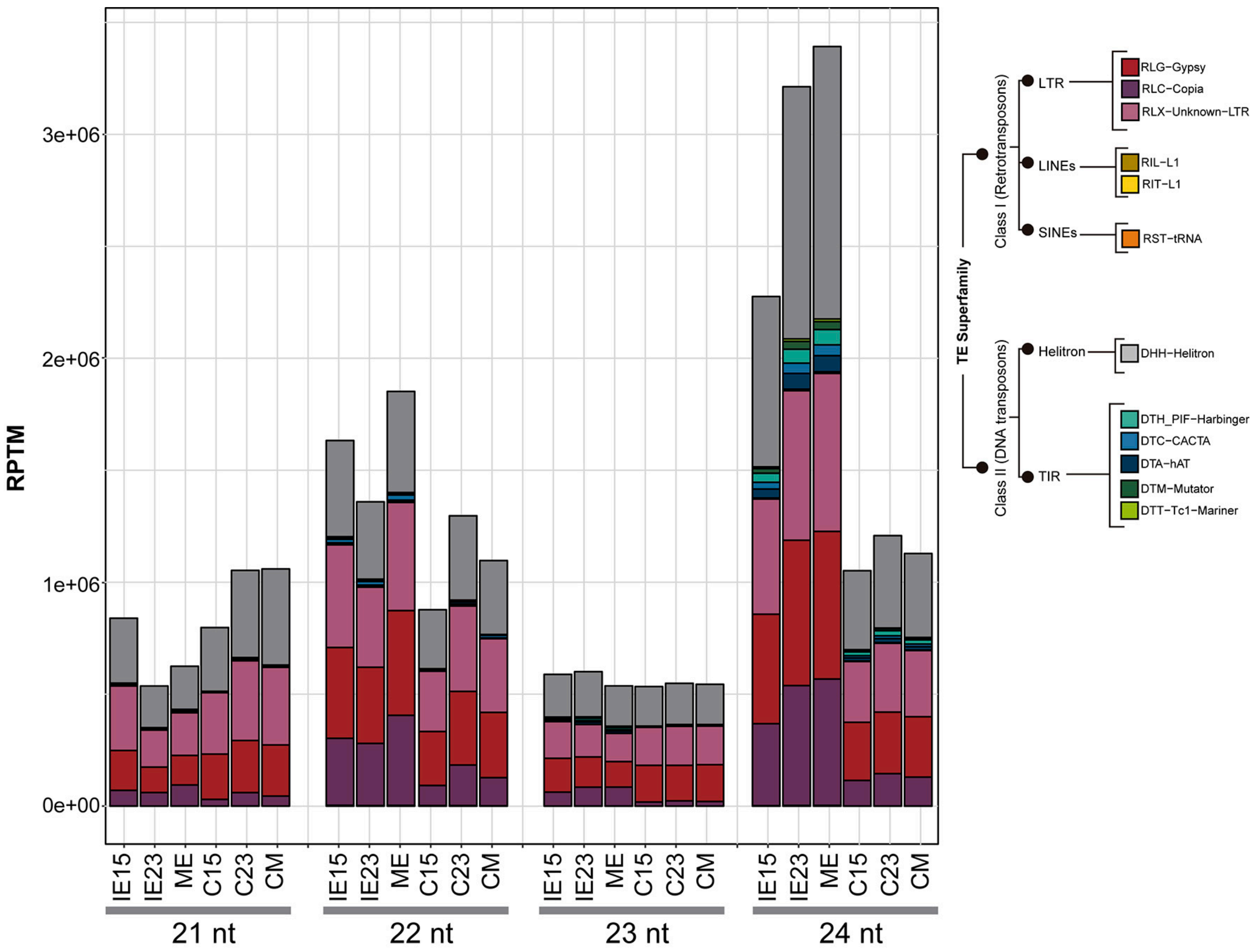

Fig. S9 Distribution of hc-siRNAs along different TEs in all tissues. Abundance of normalized reads (RPTM, Reads per ten million) that matched to major Transposable Element (TE) Superfamilies, separated by sizes (21 to 24 nt). IE15: Immature Embryos 15 days after pollination (DAP); IE23: Immature Embryos 23 DAP; ME: Mature Embryos; C15, C23 and CM: Tissues one month after induction of Somatic Embryogenesis (SE) obtained from IE15, IE23, and ME, respectively.

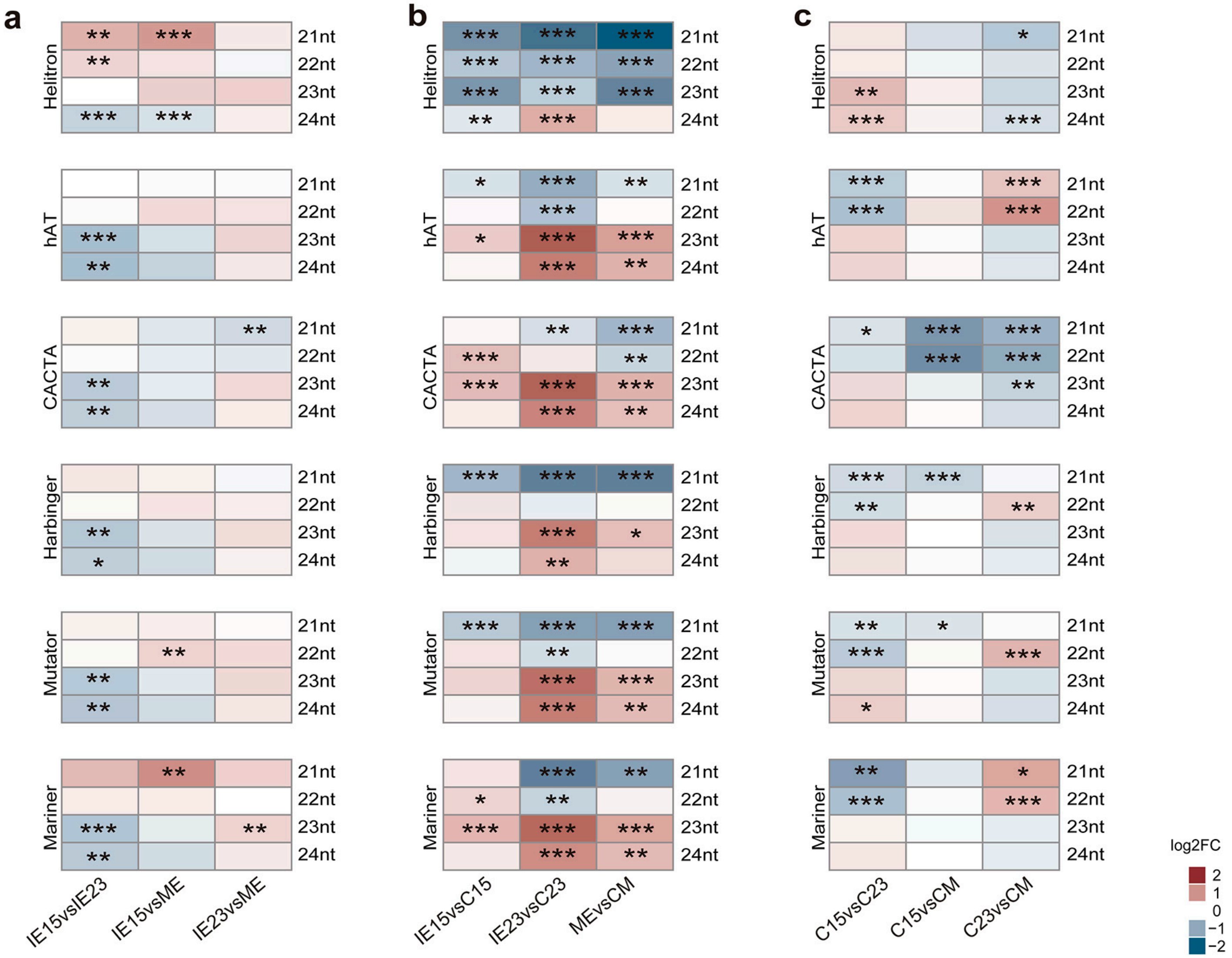

Fig. S10 Differential accumulation of hc-siRNAs mapped to DNA TEs during callus induction from maize embryos collected at different developmental stages. (a) DE between zygotic embryos developmental stages (IE15, IE23 and ME). Comparisons were done for IE15 vs. IE23, IE15 vs. ME and IE23 vs. ME. (b) DE during the induction of Somatic Embryogenesis. Comparisons were done for IE15 vs. C15, IE23 vs. C23 and ME vs. CM. (c) DE in callus tissues obtained from developmentally distinct embryos at one month of induction. Comparisons were done for C15 vs. C23, C15 vs. CM and C23 vs. CM. Values of DE were expressed as the log2 Fold Change (log2FC) and significant values indicated as follows: (*) $p<0.05$; (**) $p<0.01$; (***) $p<0.001$.

Supplementary Methods

To identify the canonical miRNA sequences corresponding to the *Zea mays* mature miRNAs from miRBase 22 (Griffiths-Jones, 2010), as well as isomiR sequences (those shifted by +/- 2 nt at the 5´or 3´ end) and those not matching to known miRNA sequences, libraries were uploaded to the Meyers lab's "Next-Gen Sequence Database" (Nakano, 2006). This facilitated our analyses by rapidly identifying these miRNAs. The final analysis included the total reads of all miRNA isoforms corresponding to the same miRNA family.

For tasiRNAs, the Phasing Analysis Window (21 nt cycle) from the Next-Gen Sequence Database was used to obtain reads mapping to the nine *TAS3* loci reported in maize (Dotto *et al.*, 2014; Xia *et al.*, 2017). Based on the sequence and the genomic location, the 21 nt sequences were filtered to obtain the total reads of eleven mature tasiARFs sequences (including those shifted by +/- 2 nt at the 5´or 3´ end).

For siRNAs mapped to transposons, the tag-count files of 21-24 nt were tested against the maize transposable element (TE) sequences, using the transposon annotation gff3 file (B73v4.TE.filtered.gff3) retrieved from the Ensembl maize annotation files (Aken *et al.*, 2016). We separated TE-mapped reads into twelve TE superfamilies from two classes: Retrotransposons (RIL, RIT, Copia, Gypsy, Unknown-LTR, tRNA) and DNA transposons (Helitron, PIF-Harbinger, CACTA, hAT, Mutator, Mariner). Since many reads matched to multiple TE superfamily, we performed a weighted normalization by the number of mapping TE superfamily (Bousios *et al.*, 2017).

Medium Composition for Callus Induction and Proliferation

N6I (Induction Medium)

N6 Salts (Chu *et al,* 1975); Murashige and Skoog Vitamin Solution (Sigma-Aldrich); 2,4-Dichlorophenoxyacetic acid (2,4-D; 2 mg/L); Adenine (10 mg/L); Proline (2.76 g/L); hydrolysed casein (200 mg/L), Sucrose (30 g/L) and Gelzan™ (Sigma-Aldrich; 3.3 g/L).

N6P (Proliferation Medium)

N6 Salts (Chu *et al,* 1975); Murashige and Skoog Vitamin Solution (Sigma-Aldrich); 2,4-D (2 mg/L); Adenine (10 mg/L); Proline (2.76 g/L); Kinetin (6-furfurylaminopurine; 0.1 mg/mL); hydrolysed casein (200 mg/L), Sucrose (30 g/L) and Gelzan™ (Sigma-Aldrich; 3.3 g/L).

Oligonucleotides used as primers in this study

ID	Orientation	SEQUENCE (5'->3')
U6 snRNA	Primer Stem Loop	GTGCAGGGTCCGAGGTTTTGGACCATTTCTCGAT
	Forward Primer	GGAACGATACAGAGAAGATTAGCA
zma-miR156	Primer Stem Loop	GTCGTATCCAGTGCAGGGTCCGAGGTATTCGCACTGGA TACGACGTGCTC
	Forward Primer	TGCTCGTGACAGAAGAGAGT
zma-miR160	Primer Stem Loop	GTCGTATCCAGTGCAGGGTCCGAGGTATTCGCACTGGA TACGACTGGCAT
	Forward Primer	TTTGCCTGGCTCCCTGT
zma-miR164	Primer Stem Loop	GTCGTATCCAGTGCAGGGTCCGAGGTATTCGCACTGGA TACGACTGCACG
	Forward Primer	CTACTGGAGAAGCAGGGCA
zma-miR166	Primer Stem Loop	GTCGTATCCAGTGCAGGGTCCGAGGTATTCGCACTGGA TACGACGGGAAT
	Forward Primer	CGTCGCTCGGACCAGGCTTCA
zma-miR390	Primer Stem Loop	GTCGTATCCAGTGCAGGGTCCGAGGTATTCGCACTGGA TACGACGGCGCT
	Forward Primer	TCTGCGAAGCTCAGGAGGGAT
tasiARFbD6	Primer Stem Loop	GTCGTATCCAGTGCAGGGTCCGAGGTATTCGCACTGGA TACGACAAAGGT
	Forward Primer	CGGCGCTCTTGACCTTGC
Universal Reverse		GTGCAGGGTCCGAGGTA
SBP23 (GRMZM2G126018_T01)	Forward	ACACCAACGCGATGAATTGG
	Reverse	ACCCTGAAAAACCAGAACGG
ARF17 (GRMZM2G159399_T01)	Forward	TTTCTCGGACATCGCTCCTG
	Reverse	CCTTGGATATACGGGGCGTC
ARF19 (AC207656.3_FGT002)	Forward	TCCCACTGTACCCGGAGCTT
	Reverse	GCATGCCTGGCTCCCTGTAT
CUC2 (GRMZM2G393433_T01)	Forward	TTCGCTGCACTACATGGTTG
	Reverse	AACGACGACCCAGTCACTTAC
RDL1 (GRMZM2G109987_T01)	Forward	GCGATTGCAGAGGAGACCTT
	Reverse	TGGCCACGATACCAACTGAA
ARF11 (GRMZM2G056120_T01)	Forward	GCACAAAAGTGTTTTTCACATTTC
	Reverse	TGTAAGTTGATCCTTGCTCCAATA
ARF24 (GRMZM2G030710_T01)	Forward	CTTCCCCATGTTAATCCAGACTAC
	Reverse	CAGCAGCATTGTCATGAGTTCTAT
Tas3g (GRMZM2G082055)	Forward	ACCTATTCACCACCGCTGTC
	Reverse	TGCGAGAGTGTTCCAAGCTC
Tas3b (GRMZM2G020468)	Forward	CTTGACCTTGTAAGACCCAACTCTA
	Reverse	TGTTTGTCTCATGCCTCACTCTAT
18S (XM_020546348.1)	Forward	TCCTATTGTTGGCCTTCGG
	Reverse	TCCTTGGCAAATGCTTTCGC
